# Childhood pneumonia in humanitarian emergencies in low- and middle-income countries: A systematic scoping review

**DOI:** 10.7189/jogh.12.10001

**Published:** 2022-04-09

**Authors:** Sally Jiasi Chen, Patrick JB Walker, Kim Mulholland, Hamish R Graham, ARI Review group, Trevor Duke, Hamish Graham, Steve Graham, Amy Gray, Amanda Gwee, Claire von Mollendorf, Kim Mulholland, Fiona Russell, Maeve Hume-Nixon, Saniya Kazi, Priya Kevat, Eleanor Neal, Cattram Nguyen, Alicia Quach, Rita Reyburn, Kathleen Ryan, Patrick Walker, Chris Wilkes, Poh Chua, Yasir Bin Nisar, Jonathon Simon, Wilson Were

**Affiliations:** Centre for International Child Health, Murdoch Children’s Research Institute, University of Melbourne, Royal Children’s Hospital, Parkville, Victoria, Australia

## Abstract

**Background:**

Humanitarian emergencies increase many risk factors for pneumonia, including disruption to food, water and sanitation, and basic health services. This review describes pneumonia morbidity and mortality among children and adolescents affected by humanitarian emergencies.

**Methods:**

We searched MEDLINE, EMBASE, and PubMed databases for publications reporting pneumonia morbidity or mortality among children aged 1 month to 17 years in humanitarian emergencies (eg, natural disaster, armed conflict, displacement) in low- and middle-income countries (LMICs).

**Results:**

We included 23 papers published between January 2000 and July 2021 from 23 countries, involving refugee/displaced persons camps (n = 5), other conflict settings (n = 14), and natural disaster (n = 3). Population pneumonia incidence was high for children under 5 years of age (73 to 146 episodes per 100 patient-years); 6%-29% met World Health Organization (WHO) criteria for severe pneumonia requiring admission. Pneumonia accounted for 13%-34% of child and adolescent presentations to camp health facilities, 7%-48% of presentations and admissions to health facilities in other conflict settings, and 12%-22% of admissions to hospitals following natural disasters. Pneumonia related deaths accounted for 7%-30% of child and adolescent deaths in hospital, though case-fatality rates varied greatly (0.5%-17.2%). The risk for pneumonia was greater for children who are: recently displaced, living in crowded settings (particularly large camps), with deficient water and sanitation facilities, and those who are malnourished.

**Conclusion:**

Pneumonia is a leading cause of morbidity and mortality in children and adolescents affected by humanitarian emergencies. Future research should address population-based pneumonia burden, particularly for older children and adolescents, and describe contextual factors to allow for more meaningful interpretation and guide interventions.

Pneumonia is the single leading infectious cause of death in children worldwide, causing 15% of all under-5 deaths in 2018 [1]. Although there has been considerable progress since 2000, pneumonia morbidity and mortality remains high and inequitably distributed, with deaths from pneumonia concentrated in Sub-Saharan Africa, South Asia, and South-East Asia [1].

Humanitarian emergencies are natural or anthropogenic events that lead to population displacement or morbidity and mortality in excess of expected trends [2]. Increased morbidity and mortality in humanitarian emergency contexts is mainly caused by indirect effects of the crisis, stemming from factors such as the breakdown of health systems, food insecurity, poor access to clean water and sanitation facilities, displacement into overcrowded makeshift settlements, missed vaccination opportunities, and air pollution, as well as outbreaks of infectious diseases[2,3].These factors directly impact on the major pillars for addressing childhood pneumonia described in the World Health Organization (WHO) and UNICEF’s Global Action Plan for Prevention and Control of Pneumonia and Diarrhoea (GAPPD) framework [4].

However, while pneumonia is a leading cause of child deaths and humanitarian emergencies exacerbate underlying risk factors, few studies have documented pneumonia incidence or risk factors for children affected by humanitarian emergencies. A previous review conducted in 2010 by Bellos and colleagues found that acute respiratory infections (ARIs) contributed to excess morbidity and mortality in humanitarian emergencies, accounting for 20 to 35% of all young child deaths [5]. But the authors observed marked heterogeneity between studies and were challenged by limited and poor quality data, including unclear ARI case definitions, lack of population-level data, and minimal data on children over 5 years [5]. The authors called for more research focussing on pneumonia (lower respiratory tract infection), using clear and standardised case definitions, providing population-level morbidity and mortality estimates, and better age disaggregation [5].

We conducted this systematic scoping review to (i) describe WHO-defined pneumonia burden (morbidity and mortality) and risk among children and adolescents affected by humanitarian emergencies, including variation between contexts, and (ii) map the literature on pneumonia in children affected by humanitarian emergencies, including data on aetiology, prevention and treatment.

## METHODS

We developed the protocol for this review using PRISMA-P, PRISMA-ScR, and PROSPERO guidelines but were not able to register it as PROSPERO does not accept the registration of scoping review protocols [6,7].

We searched MEDLINE, EMBASE, and PubMed (for studies not yet indexed in MEDLINE) for studies published between January 2000 and July 2021 examining pneumonia in children and adolescents affected by humanitarian emergencies. We ran the search run in July 2020 and updated it on 14 July 2021 to capture more recent publications (example search strategy included in (details in Text S1 and S2 of the **Online Supplemental Document**). We also searched websites for unpublished reports (eg, from UN and humanitarian agencies) and contacted individuals known to be working in this area.

Two researchers (SC and PW) independently screened titles/abstract, and then full-text articles, for inclusion, with discrepancy resolved by consensus or third researcher (HG). We included studies that presented original data on pneumonia burden (eg, incidence, proportional morbidity, proportional mortality, case-fatality rate) in children and adolescents aged 1 month to 17 years of age affected by a humanitarian emergency (eg, natural disasters, forced displacement, refugee camps, famines, and armed conflict/war) in LMICs. We excluded studies focusing on a particular cause of respiratory infection (eg, influenza), respiratory epidemics (eg, SARS, MERS, COVID-19), or refugee or asylum seeker populations resettled in high-income countries.

One researcher (either SC or PW) extracted data from each study into an electronic data form, including data on study design, setting, type of crisis, pneumonia definition, morbidity and mortality, and other contextual factors. We used the Effective Public Healthcare Panacea Project (EPHPP) Quality Assessment Tool for Quantitative Studies to assess study quality but did not exclude any studies based on quality.[8,9] The EPHPP quality assessment tool is a comprehensive tool developed for assessing risk of bias for a broad range of quantitative studies and was preferred over other risk of bias tools intended for randomised (eg, Cochrane Risk of Bias tool) or non-randomised intervention studies (eg, ROBINS-I) [8-10].

In accordance with scoping review methodology [11-13], we mapped the existing literature using numerical summaries of included studies and described pneumonia incidence, proportional morbidity, mortality, and other key findings and gaps through narrative synthesis. Where possible, we stratified relevant data and findings by age and setting.

**Ethics approval**: Not required.

## RESULTS

**Figure 1:**
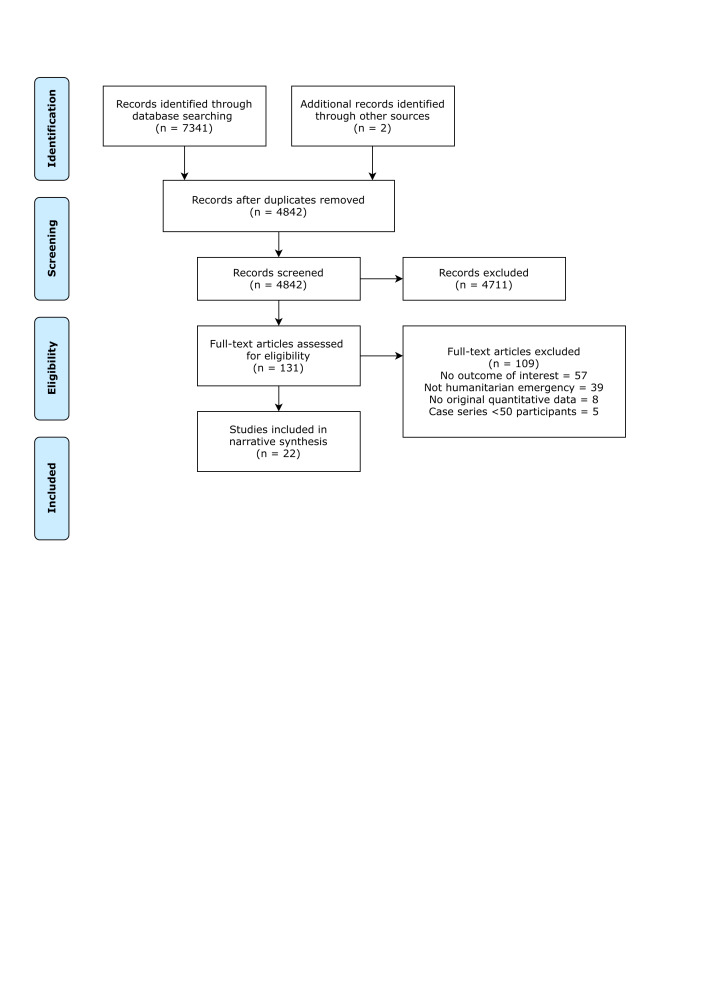
PRISMA 2009 flow diagram of literature search.

Our database searches returned 4842 unique citations; two additional studies were identified through other sources. We reviewed 131 potentially relevant studies, including 22 studies for qualitative synthesis (**Figure 1**, **Table 1** and **Table 2**).

**Table 1:** Characteristics of included studies (n = 22) on childhood pneumonia in humanitarian emergencies in low- and middle-income countries from the year 2000–2021

Study characteristics*		Number (%)
**World Bank income category**	LIC	15 (68%)
	LMIC	5 (23%)
	UMIC	4 (18%)
**WHO region**	EMRO	10 (45%)
	SEARO	6 (27%)
	AFRO	6 (27%)
	WPRO	2 (9%)
	EURO	0 (0%)
	PAHO	0 (0%)
**Countries**	**Single country studies**	21 (95%)
	Afghanistan	6 (27%)
	Thailand	3 (14%)
	Democratic Republic of Congo	2 (9%)
	Philippines	2 (9%)
	Syria	2 (9%)
	Bangladesh	1 (5%)
	Central African Republic	1 (5%)
	Guinea-Bissau	1 (5%)
	Liberia	1 (5%)
	Nepal	1 (5%)
	Somalia	1 (5%)
	**Multiple country studies**	1 (5%)†
**Age of participants**	Infant 1-12 mo	22 (100%)
	Children 1-4 y	21 (95%)
	Older children 5-9 y	9 (41%)
	Adolescents 10-17 y	9 (41%)
**Healthcare setting**	Inpatient	12 (55%)
	Outpatient	4 (20%)
	Community	6 (27%)
**Type of humanitarian emergency**	Armed conflict/war	14 (64%)
	Refugees/IDPs	5 (23%)
	Natural disaster	3 (14%)
	-Typhoon	2 (9%)
	-Earthquake	1 (5%)
	-Drought, Famine, Flood	0 (0%)
**Focus on pneumonia**	Pneumonia-focused	8 (36%)
	Pneumonia studied among multiple diseases	14 (64%)
**Study design**	Randomised trial	2 (9%)
	Other interventional studies	0 (0%)
	Case-control study	0 (0%)
	Cross-sectional study	13 (59%)
	Cohort study	4 (18%)
	Surveillance study	2 (9%)
	Before and after study	2 (9%)
**Key outcomes**	Incidence	5 (23%)
	Proportional morbidity	13 (59%)
	Proportional mortality	4 (18%)
	Case fatality rate	8 (36%)

LIC – low-income country, LMIC – lower-middle-income country, UMIC – upper-middle-income country, EMRO – Eastern Mediterranean Regional Office, SEARO – South-East Regional Office, AFRO – African Regional Office; WPRO – Western Pacific Regional Office, EURO – European Office, PAHO – Regional Office for the Americas,UNHCR – United Nations High-Commissioner for Refugees

*Categories are not mutually exclusive.

†Hershey et al (2011) study includes data on UNHCR refugee camps in the following 16 countries: Bangladesh, Burundi, Cameroon, Chad, Democratic Republic of Congo, Djibouti, Ethiopia, Kenya, Namibia, Nepal, Rwanda, Sudan, Tanzania, Thailand, Uganda and Yemen.

**Table 2:** Characteristics of included studies (n = 22) on childhood pneumonia in humanitarian emergencies in low- and middle-income countries from January 2000 to July 2021

Study	Country (region*)	Study type	Setting (data collection period)	Study population	Pneumonia case definition (as reported)	Outcomes reported	Study quality
Anwar et al (2017) [19]	Afghanistan (EMRO)	Cross-sectional	Conflict-affected population. Protracted, complex emergency (2005-2013).	13 404 322 new visits of children aged <5 y old to Basic Package Health Services facilities in Afghanistan. National study using HMIS data.	Healthcare worker diagnosis during routine care. Case definition based on Afghanistan IMCI guidelines (pneumonia = cough or difficult breathing, AND fast breathing for age).	Proportional morbidity for pneumonia and other acute conditions.	W
Bernasconi et al (2018) [21]	Afghanistan (EMRO)	Before and after study	Conflict-affected population. Protracted, complex emergency (2005-2013).	8646 children aged 2-59 mo old who presented to one of three Basic Health Centres (BHCs) in Afghanistan (599 consultations in baseline survey, 8047 consultations after implementation of ALMANACH).	Healthcare worker diagnosis during routine care. Case definition based on Afghanistan IMCI guidelines.	Proportional morbidity for pneumonia and other acute conditions. Assess HCW care against IMCI standards.	W
Birindwa et al (2020) [27]	Democratic Republic of the Congo (AFRO)	Before and after study	Conflict-affected population. Protracted, complex emergency (2010-2015).	2007 children aged 2-59 mo old admitted to one of two general referral hospitals or two district hospitals with a diagnosis of ALRI and who completed prescribed inpatient treatment.	Doctor diagnosis during routine care. Pneumonia not explicitly defined, but reported as “acute lower respiratory tract infection, ALRI”.	Proportional morbidity for pneumonia and case fatality rate.	W
Chang et al (2016) [31]	Philippines (WPRO)	Cross-sectional	Typhoon-affected population. Pre-crisis, crisis, and stabilisation phases. (Sep 2013-Feb 2014).	857 children aged 0-17 y old admitted to a level 2 hospital in Philippines at the time of Typhoon Haiyan (8 Nov 2013).	Doctor diagnosis during routine care. “Pneumonia” reported but not explicitly defined.	Proportional morbidity for pneumonia and other acute conditions.	W
Clarke-Deelder et al (2019) [20]	Democratic Republic of the Congo (AFRO)	Cross-sectional	Conflict-affected population. Protracted, complex emergency (Jun 2015-Mar 2016).	366 children aged 2 mo – 5 y with IMCI classified severe disease (includes severe febrile disease, severe pneumonia, and severe dehydration) presenting to 266 health centres and 80 hospitals (randomly selected from all government facilities nationally).	Trained clinical data collectors via direct observation of consultations, using WHO IMCI classification 2014 for severe pneumonia (cough OR difficulty breathing AND any general danger sign).	Proportional morbidity for severe pneumonia, severe febrile disease, and diarrhoea with severe dehydration). Assess HCW care against IMCI standards.	W
Giri et al (2018) [32]	Nepal (SEARO)	Cross-sectional	Earthquake-affected population. Post-crisis phase (May-Aug 2015).	1057 children aged <14 y old admitted to the general pediatrics department of a Kathmandu tertiary hospital with non-traumatic or non-surgical illnesses, in a 15-week period following the 7.8 magnitude Nepal earthquake on 25 April 2015.	Doctor diagnosis during routine care. “Pneumonia” reported but not explicitly defined.	Proportional morbidity.	W
Hershey et al (2011) [17]	16 countries^†^ (AFRO, SEARO, EMRO)	Cross-sectional	Refugees living in refugee camps in 16 countries. Mix of acute crisis, post-crisis, protracted complex emergency settings (Jan 200-Feb 2010).	Children aged <5 y attending health facilities in 90 UNHCR refugee camps in 16 countries. Uses UNHCR HMIS data.	Healthcare worker diagnosis during routine care. UNHCR HIS case definition of pneumonia in children 2 mo to 5 y of age: cough or difficulty breathing and breathing faster than 50 breaths/min (2-12 mo of age) or breathing faster than 40 breaths/min (1-5 y of age).	Incidence, proportional morbidity, and proportional mortality, for pneumonia, malaria, and diarrhoea.	W
Huerga et al (2009) [30]	Liberia (AFRO)	Cross-sectional	Conflict-affected population during post-crisis phases (Jan-Jul 2005).	5137 patients admitted to a referral hospital in Monrovia, Liberia 1 y after the civil war ended (including 1509 children aged 0 to 14 y old hospitalised in the paediatric ward).	Doctor diagnosis during routine care using MSF clinical guidelines. Pneumonia not explicitly defined and reported as “respiratory infection”.	Case fatality rate and proportional mortality.	W
Manaseki-Holland et al (2012) [16]	Afghanistan (EMRO)	Randomised controlled trial	Conflict-affected population. Protracted, complex emergency (Dec 2008-Jun 2009).	3046 children aged 1-11 mo living in Afghanistan enrolled to community-based RCT.	Clinical data collectors using WHO IMCI clinical definition of pneumonia (cough plus increased respiratory rate for age), severe pneumonia (cough + chest indrawing), very severe pneumonia (cough plus any danger sign - not feeding, convulsions, vomiting, lethargic or unconscious, stridor in a calm child). “Confirmed” pneumonia determined using CXR reported by trained radiologist using WHO interpretation standards.	Incidence, incidence of death from pneumonia.	S
Manaseki-Holland et al (2010) [35]	Afghanistan (EMRO)	Randomised controlled trial	Conflict-affected population. Protracted, complex emergency (Feb-May 2007).	453 children aged 1-36 mo diagnosed with IMCI-classified pneumonia at a teaching hospital in Kabul, Afghanistan.	Doctor diagnosis using WHO clinical definitions for pneumonia (age-specific tachypnoea WITHOUT wheeze), severe pneumonia (pneumonia PLUS chest indrawing), and very severe pneumonia (pneumonia PLUS any danger signs - central cyanosis, severe respiratory distress, inability to drink, convulsions, vomiting).	Case fatality rate.	S
Meiqari et al (2018) [28]	Syria (EMRO)	Cross-sectional	Conflict-affected population. Acute and protracted, complex emergency (2013-2016).	Children aged <18 y old using MSF-OCA health facilities in northern Syria (4672 in-patient admissions, and 27 742 out-patient consultations).	Doctor/health care worker diagnosis using MSF clinical guidelines. Pneumonia not explicitly defined, but reported as “acute lower respiratory tract infection, ALRI”.	Proportional morbidity, case fatality rate.	W
Ngoy et al (2013) [23]	Somalia (EMRO)	Cross-sectional	Conflict-affected population. Protracted, complex emergency (2010-2011).	6211 children aged <15 y admitted to the paediatric ward of Istalin hospital, in Guriel district, Somalia.	Doctor diagnosis using MSF clinical guidelines. Pneumonia not explicitly defined, but reported as “lower respiratory tract infection, LRTI”.	Proportional morbidity, case fatality rate, proportional mortality.	W
Rasooly et al (2020) [24]	Afghanistan (EMRO)	Cross-sectional	Conflict-affected population. Protracted, complex emergency (Jan-Feb 2018).	752 children aged 2-59 mo admitted to Balkh Regional Hospital with pneumonia or severe pneumonia.	Doctor diagnosis during routine care. Pneumonia and severe pneumonia were reported but not explicitly defined.	Case fatality rate.	N/A^‡^
Robinson et al (2021) [34]	Central African Republic (AFRO)	Cross-sectional	Conflict-affected population. Protracted, complex emergency (Mar-Apr 2020).	591 households (4272 household members) in Ouaka prefecture, Central African Republic who participated in a population-based, two-stage cluster survey.	Verbal reporting by household head. Respiratory infection was reported but not explicitly defined.	Proportional mortality.	W
Sodemann et al (2004) [22]	Guinea-Bissau (AFRO)	Cross-sectional and surveillance	Conflict-affected population, acute/subacute crisis (Jun 1997-Jun 1999).	**War cohort:** 2947 children hospitalized at the paediatric ward between 7 June 1998 and 6 June 1999. **Peace cohort:** 6449 children hospitalized in the preceding 12-mo period from 7 June 1997 to 6 June 1998.	Doctor diagnosis during routine care. Pneumonia was reported but not explicitly defined.	Proportional morbidity, case fatality rate.	W
Summers et al (2018) [14]	Bangladesh (SEARO)	Cross-sectional	Conflict-affected refugees living in refugee camps, including recently displaced and long-stayers (Oct-Nov 2017).	1827 children aged 6-59 mo old in a Rohingya refugee population living in Kutupalong refugee camp, makeshift settlements, and Nayapara refugee camp in Cox’s Bazar, Bangladesh.	Caregiver report via household survey. Acute respiratory infection (ARI) was defined as cough with rapid breathing or difficulty breathing and a fever within the 2 weeks preceding the survey.	Incidence.	W
Turner, C. et al (2012) [36]	Thailand (SEARO)	Cohort study	Refugees living in refugee camp (Sep 2007-Sep 2010).	A birth cohort of 955 refugee infants recruited from antenatal clinics in Maela refugee camp, Thailand, followed until two years of age.	Clinical data collector during routine visits, and health care worker during illness visits, using WHO case definitions for clinical pneumonia (cough or difficulty breathing and age-specific tachypnoea), severe pneumonia (cough or difficulty breathing plus chest indrawing, very severe pneumonia (severe pneumonia plus cyanosis or inability to suck). CXRs interpreted by 2 clinicians using WHO criteria.	Incidence.	M
Turner, C. et al (2013) [15]	Thailand (SEARO)	Cohort study	Refugees living in refugee camp (Sep 2007-Sep 2010).	A birth cohort of 955 refugee infants recruited from antenatal clinics in Maela refugee camp, Thailand, followed until two years of age.	Clinical data collector during routine visits, and health care worker during illness visits, using WHO case definitions for clinical pneumonia (cough or difficulty breathing and age-specific tachypnoea), severe pneumonia (cough or difficulty breathing plus chest indrawing, very severe pneumonia (severe pneumonia plus cyanosis or inability to suck). CXRs interpreted by 2 clinicians using WHO criteria.	Incidence, incidence of death from pneumonia.	M
Turner, P. et al (2013) [25]	Thailand (SEARO)	Surveillance study	Refugees living in refugee camps (Apr 2009-Sep 2011).	698 refugees living in Maela refugee camp, Thailand admitted to a humanitarian organisation hospital with pneumonia.	WHO criteria for clinical pneumonia U5 (cough or difficulty breathing AND age-specific tachypnoea), severe pneumonia (pneumonia with chest indrawing), and very severe pneumonia (pneumonia with one of the following: difficulty feeding, lethargy, convulsions, severe respiratory distress, loss of consciousness, or central cyanosis. British Thoracic Society case definition in children ≥5y for clinical pneumonia (history of fever OR fever ≥38°C PLUS cough OR difficulty breathing PLUS abnormal chest examination).	Proportional morbidity.	M
van Berlaer et al (2019) [29]	Philippines (WPRO)	Cross-sectional study	Typhoon-affected population, from 1 week post-event (16-20 Nov 2013).	1267 patients presenting to a field hospital in the Philippines 1 week after typhoon Haiyan (8 Nov 2013).	Doctor diagnosis during routine care based on clinical features: lower respiratory tract infection, LRTI (“dyspnoea, tachypnoea, signs of lower ARI”).	Proportional morbidity.	W
Van Berlaer et al (2017) [18]	Syria (EMRO)	Cross-sectional study	Conflict-affected population. Protracted, complex emergency 4 y after the start of the Syrian civil war (21-22 May 2015).	1002 children aged <15 y old in four northern Syrian governorate districts (Aleppo, Idleb, Hamah, and Lattakia).	Clinical data collector during community and facility-based survey. Lower respiratory tract infection (LRTI) case description: “dyspnoea, and raised respiratory rate, signs of lower ARI”.	Proportional morbidity.	W
Zabihullah et al (2017) [26]	Afghanistan (EMRO)	Cohort study	Conflict-affected population. Protracted, complex emergency (Dec 2012-Mar 2013).	639 children <5 y of age who met the WHO criteria for clinical pneumonia at the time of admission to the paediatric department of a regional refreral hospital in Mazar-e-Sharif, Afghanistan.	Doctor diagnosis on admission using standardised data collection form and WHO criteria for clinical pneumonia (cough or difficulty breathing AND age-specific tachypnoea), severe pneumonia (pneumonia with chest indrawing), and very severe pneumonia (pneumonia with one of the following: difficulty feeding, lethargy, convulsions, severe respiratory distress, loss of consciousness, or central cyanosis.	Case fatality rate, aetiology.	M

EMRO – Eastern Mediterranean Regional Office, SEARO – South-East Regional Office, AFRO – African Regional Office, WPRO – Western Pacific Regional Office; EURO, European Office, PAHO – Regional Office for the Americas, IMCI – Integrated Management of Childhood Illness, HCW – health care worker, BPHS – Basic Package Health Services, UNHCR – United Nations High Commissioner for Refugees, LRTI – lower respiratory tract infection, ARI – acute respiratory infection; CXR – chest x-ray, mo – month, y - year

*WHO Region.

†Hershey et al (2011) [17] study included data on UNHCR refugee camps in the following 16 countries: Bangladesh, Burundi, Cameroon, Chad, Democratic Republic of Congo, Djibouti, Ethiopia, Kenya, Namibia, Nepal, Rwanda, Sudan, Tanzania, Thailand, Uganda and Yemen.

‡N/A, not applicable. Unable to assess quality due to limited data in conference abstract (no related full manuscript was available).

### Study characteristics

Studies included data from 23 countries, including one paper that reported data from 16 countries (Table S1 in the **Online Supplemental Document**, **Table 1**). Two-thirds (14/22, 64%) of studies were published since 2015. Study duration ranged from a few days to 9 years (median 6 months, interquartile range 3 to 34 months).

Fourteen (64%) studies included populations affected by armed conflict, five (23%) studies reported on displaced persons (all living in refugee camps), and three (14%) studies reported on populations affected by a natural disaster.

Almost all studies reported data on children aged under 5 years old (21/22, 95%) but less than half (9/22, 41%) contained data relating to children 5-17 years old. Study quality was generally low; 6/22 (27%) studies scored moderate (n = 4) or high (n = 2) using the EPHPP quality assessment tool (Text S3 in the **Online Supplemental Document**).

### Pneumonia incidence

**Figure 2:**
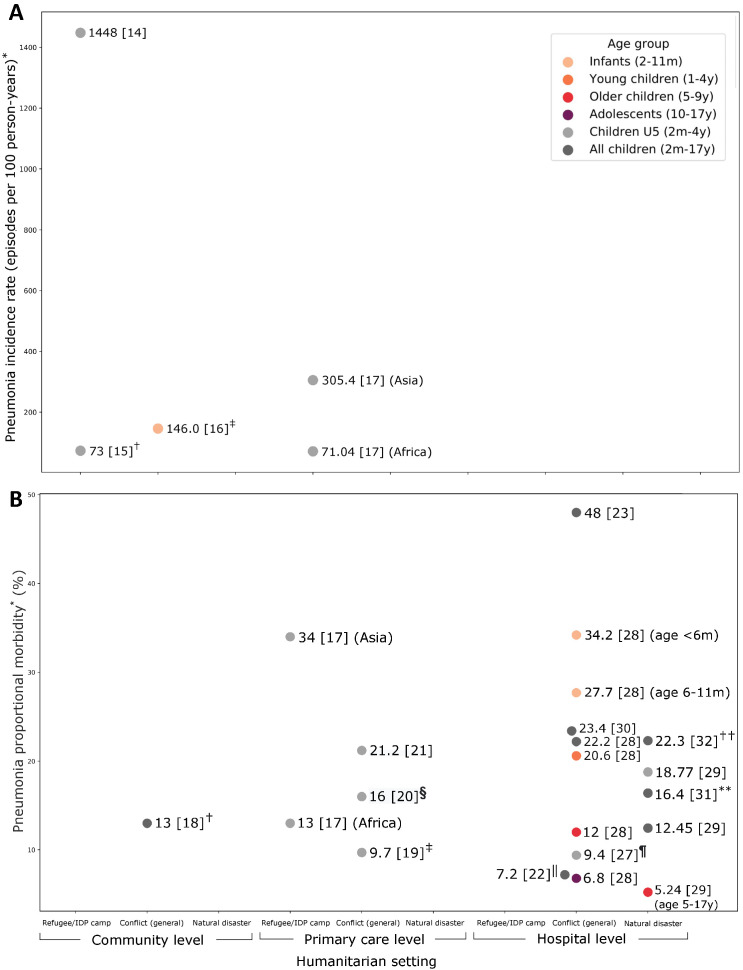
Pneumonia incidence (**panel A**) and proportional morbidity (**panel B**) among children in humanitarian emergencies in low- and middle-income countries. **Panel A.** IDP – internally displaced persons; U5 – children under 5 years of age. *Pneumonia incidence rates have been converted to episodes per 1000 person-years to allow comparison between studies. †Pneumonia classified using pre-2014 WHO pneumonia definitions. Incidence rates for sub-classifications of pneumonia were as follows: 50 (non-severe), 15 (severe), 6 (very severe) and 22 (radiological end-point). ‡Pneumonia classified using WHO pneumonia and severe pneumonia categories. **Panel B.** Pneumonia proportional morbidity among children in humanitarian emergencies in low- and middle-income countries. IDP - internally displaced persons, U5 – children under 5 years of age. *Proportional morbidity – pneumonia cases as a proportion of all paediatric cases in community, presenting to outpatient facilities, or admitted to hospital, unless otherwise specified. †Van Berlaer reported for pneumonia as a primary diagnosis (9.0%), or as any diagnosis (13%). ‡Anwar also reported pneumonia morbidity estimates in three-year intervals: 12.2% (2005-2007), 9.9% (2008-2010), and 8.6% (2011-2013). §Severe pneumonia, % of all paediatric outpatients. ‖Sodemann also reported a pneumonia morbidity estimate of 10.8% in the pre-conflict period. ¶Birindwa also reported pneumonia morbidity rates of 12.2% pre-PCV13 and 7.1% post-PCV13. **Pneumonia, % of paediatric medical admissions during the period of impact of an earthquake. Pneumonia morbidity rates pre-impact and post-impact were reported as 22.0% and 19.4% respectively. ††Pneumonia, % of paediatric medical admissions.

Four studies reported pneumonia incidence for young children (infants or children U5), including three from refugee camp settings with no data for older children or adolescents (**Figure 2**, Panel A, Table S1 in the **Online Supplemental Document**).

The highest pneumonia incidence was reported by Summers et al using household survey data from Rohingya families in refugee camps in Cox’s Bazar, Bangladesh, reporting that 54% of children under five years of age (U5) had WHO-defined pneumonia in the previous 2 weeks ( ~ 1448 per 100 person-years) [14]. However, this study relied on caregiver recall over a short time period and is likely to have a high risk of bias from selective reporting. This study found that the risk of pneumonia was higher in unregistered (newer arrivals) vs registered camp residents in univariate analysis (*P* = 0.002), and around two-thirds of caregivers reported seeking care from formal health services [14].

Two higher quality studies also reported population-level pneumonia incidence. Turner et al reported data from a prospective birth cohort of children under 2 years of age living in a long-term refugee camp in Thailand, finding pneumonia incidence of 73 and 22 per 100 person-years for clinical and radiological end-point pneumonia, respectively (21% met WHO criteria for chest-indrawing pneumonia, 8% pneumonia with danger signs) [15]. Adjusted analysis suggested that pneumonia diagnosis was associated with crowded housing, cookstoves nearby bed, and lower maternal age [15]. Manaseki-Holland et al. reported data from a community-based randomised controlled trial (RCT) in urban Kabul, Afghanistan, during protracted civil conflict, finding pneumonia incidence of 146 per 100 person-years for infants aged 2-11 months of age followed for 6 months (7% met WHO criteria for chest-indrawing pneumonia) [16]. These studies suggested that 7%-8% of pneumonia cases identified in the community met WHO criteria (2014) for severe pneumonia requiring hospitalisation [15,16].

One multi-country study reported pneumonia incidence for children under five years in refugee camp settings in 16 countries in Asia and Africa, based on UNHCR health information system data on presentations to health facilities. The mean incidence of pneumonia was 71 per 100 child-years in African camps, and 305 in Asian camps, with wide variability between individual camps (from <40 to >400), highlighting problems with case ascertainment. Adjusted analysis suggested that the risk of pneumonia was higher in camps that were larger, had poorer access to water, and where health facilities had a higher proportion of first visits (likely representing newer arrivals) – and that geography was not a significant predictor after these factors had been accounted for (incidence rate ratio, Asia compared to Africa = 1.65, 95% confidence interval (CI) = 0.79-3.43) [17].

### Proportional morbidity attributable to pneumonia

**Figure 3:**
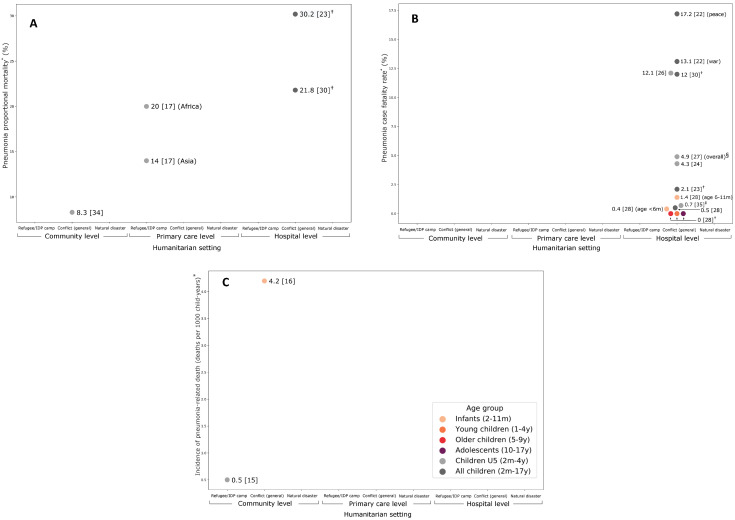
Proportional mortality (**panel A**), case fatality rate (**panel B**), pneumonia incidence of death (**panel C**) among children in humanitarian emergencies in low- and middle-income countries. **Panel A.** IDP – internally displaced persons, U5 – children under 5 years of age. *Case fatality rate – pneumonia deaths as a proportion of pneumonia cases. †Under 15 years of age. ‡1-36 months of age. §Birindwa also reported pneumonia mortality rates of 4.8% pre-PCV13 and 4.9% post-PCV13. **Panel B.** IDP – internally displaced persons, U5 – children under 5 years of age. *Estimates for incidence of pneumonia-related death have been converted to deaths per 1000 person-years to allow comparison between studies. **Panel C.** IDP – internally displaced persons, U5 – children under 5 years of age. *Proportional mortality = pneumonia deaths as a proportion of all paediatric deaths in community, or hospital, unless otherwise specified. †Under 15 years of age.

Thirteen studies reported on the proportional morbidity of childhood pneumonia, including two with data on older children or adolescents (**Figure 3**, Panel B, Table S1 in the **Online Supplemental Document**).

One study reported proportional morbidity at *population level*, using data from a community-based survey among children <15 years of age living in homes and IDP camps in northern Syria during the civil war [18]. Lower respiratory tract infection was the most common condition, reported as a primary concern for 9% of children surveyed, and a secondary concern for another 4%. Infectious disease was reported in a lower proportion of children from IDP camps than town residents (57.1% vs 65.1%), but adjusted analysis suggested that this difference was entirely explained by geographical location and age (more common in younger children and those in Aleppo) and that ‘displacement’ was not an independent predictor of infectious morbidity (odds ratio (OR) = 1.01, 95% CI = 0.71-1.44) [18].

In four studies looking at pneumonia in *outpatient settings*, pneumonia represented 12%-34% of diagnoses for children U5 [17,19-21]. No studies included older children or adolescents. Hershey et al’s study was the only study that reported proportional morbidity from a refugee camp setting, finding that pneumonia accounted for 17% of presentations across camps in 15 countries. This study found that pneumonia accounted for 34% and 13% of presentations to camps in Asia and Africa respectively, with malaria predominating in African settings [17].

Nationally representative data from conflict-affected Afghanistan showed that pneumonia cases decreased as a proportion of U5 admissions from 12.2% to 8.6% between 2005 and 2013 [19]. However, Bernasconi et al’s study of children attending primary care facilities in Kabul, Afghanistan, revealed significant under-recognition of pneumonia by health care workers finding that pneumonia represented 9.7% (39/404) of U5 presentation at baseline but 21.2% (141/665) of presentations after introduction of an electronic decision-making tool that encouraged adherence to IMCI guidelines [21].

Clarke-Deelder et al. reported data from children presenting to facilities in the Democratic Republic of Congo (DRC), finding that 189 out of 1180 included children (16%) met WHO criteria for severe pneumonia. Of these severe pneumonia cases, 52% were identified as respiratory infection by the health care worker (usually a nurse), 69% received antibiotics, but only 13% were recommended for inpatient care (admission or referral to hospital) [20].

In eight studies looking at child pneumonia in *hospital settings*, the proportion of admissions due to pneumonia ranged from 7.2% in a war-affected cohort of children in Guinea-Bissau [22] to 48% in a conflict-affected region of Somalia [23]. In hospital settings, severe and very severe pneumonia accounted for 24% to 85% of pneumonia diagnoses [24-27]. Two studies disaggregated data by age, finding that the proportion of admissions due to pneumonia decreased with increasing age [28,29].

Five hospital-based studies reported proportional morbidity in populations of children affected by conflict, with pneumonia accounting for between 7.2% to 48% of admissions (median 22.8%) [22,23,28,30]. The lowest estimate (7.2%) was reported from the aforementioned Guinea-Bissau study in which 73% of children admitted were diagnosed with malaria and mortality among those diagnosed with pneumonia was particularly high (13.1%), suggesting possible under-recognition of pneumonia [22]. This study was also the only one to compare populations before and after the onset of war, finding that 10.8% and 7.2% of admitted children had pneumonia in the war and peace cohorts respectively [22]. Three other studies from hospitals run by *Médecins Sans Frontières* (MSF) in Liberia, Syria and Somalia reported proportional morbidity attributable to pneumonia of 23%, 22%, and 48% of paediatric admissions, respectively. One before-after study from the Democratic Republic of Congo (DRC) found that the introduction of the 13-valent pneumococcal conjugate vaccine (PCV-13) reduced the proportion of U5 admissions due to pneumonia, from 12.2% to 7.1% (*P* < 0.0001) [27]. This was the only study we identified that directly assessed the efficacy of pneumonia vaccination on child pneumonia in a humanitarian setting.

Three hospital-based studies reported proportional morbidity in children affected by natural disasters, with pneumonia accounting for 12% to 22% of admissions during and after natural disasters [29,31,32]. Two studies contained data relating to the 2013 Typhoon Haiyan in the Philippines, reporting that pneumonia accounted for 12%-16% of paediatric admissions during the crisis, and around 19% of U5 admissions [29,31]. One study compared pre- and post-crisis data, finding an increase in childhood pneumonia admission numbers but a relative reduction in pneumonia proportional morbidity (22.0% to 16.4%) due to an even greater increase in diarrhoeal disease and dengue [31]. One study from a Kathmandu hospital immediately following the 2015 Nepal earthquake, found pneumonia accounted for 22% of paediatric admissions and was more common among children from affected districts compared to admissions among those from non-affected districts (26.6% vs 17.0%, *P* < 0.001, univariate analysis) [32].

While a number of these studies included older children and/or adolescents, only two studies reported disaggregated data for the older age groups. Pneumonia accounted for 7% and 12% of admissions for children aged 5-9 and 10-17 years, respectively, to Médecins Sans Frontières (MSF) facilities in Syria, and 5% of children aged 5-17 years presenting to facilities after Typhoon Haiyan in Philippines [28,29].

### Pneumonia mortality

Twelve studies reported on childhood pneumonia mortality (proportional mortality, case-fatality rates, incidence of death), including three with data on older children or adolescents (**Figure 3**, Table S2 in the **Online Supplemental Document**).

### Incidence of pneumonia-related death

Two studies reported on population-level risk of death for pneumonia for young children (age ≤2 years) (**Figure 3**, Panel A). Among a birth cohort of infants up to 2 years of age in a Thai refugee camp, pneumonia mortality was 0.5 deaths per 1000 child-years [15]. Among infants (1-11 months of age) enrolled in an RCT in urban Kabul, Afghanistan, pneumonia mortality was 4.2 per 1000 child-years [16]. For comparison, contemporaneous infant mortality ratios (IMR) were 12.2 (Thailand) and 66.5 (Afghanistan) per 1000 livebirths in 2009 [33].

### Proportional mortality attributable to pneumonia

Four studies reported proportional mortality among children and adolescents, ranging from 8% [34] to 30% of admissions (**Figure 3**, Panel B) [23]. A household mortality survey in the Central African Republic found that respiratory infection accounted for 8.3% of under-five deaths (excluding neonates) due to known causes – with most deaths attributed to fever/malaria (42%), diarrhoea (31%), and measles (8%) [34]. Huerga et al reported that pneumonia deaths accounted for 22% of all child deaths among children aged <15 years admitted to a referral hospital in Liberia, one year after the civil war had ended, with a case fatality rate (CFR) of 12% [30]. Ngoy et al found that pneumonia accounted for 30% of deaths among children aged <15 years admitted to an MSF hospital in Somalia, with CFR of 2.1% [23]. The lowest estimates for proportional mortality attributable to pneumonia were from a multi-country study using routine data from United Nations High Commissioner for Refugees (UNHCR) health facilities in refugee camps which reported pneumonia deaths accounting for 20% of U5 deaths (20% in African camps, 14% in Asian camps) [17]. However, this study recorded one-third of deaths as “other cause”, and included neonates who would have contributed disproportionately to deaths, so the contribution of pneumonia to child deaths from this data is unclear.

### Pneumonia case fatality rates

Six additional studies reported pneumonia CFR from facilities in conflict-affected settings, ranging from 0.5% [28] to 13% [22]. We found no data on refugee camp or natural disaster settings (**Figure 4**, Panel C).

The highest CFR was reported among children admitted to a tertiary hospital following the onset of civil war in Guinea-Bissau [22]. Univariate analysis showed that CFR for pneumonia (and other conditions) trended downwards after the onset of civil war (17% vs 13%, OR 0.72, 95% CI 0.46–1.13), despite higher bed occupancy and a greater proportion of deaths occurring before formal admission. Importantly, this was one of very few included studies that included data on deaths that occurred prior to formal admission (including them in CFR calculation). Feedback from healthcare workers suggested the observed reduction in case fatality in the early conflict period was due to better access to essential medications and supplies, and better support and morale of staff (who were required to reside on campus due to insecurity).(23) Similarly high CFRs were reported from urban hospitals in conflict-affected Liberia (12% in children under 15) [30], and Afghanistan (12% in children under 5) [26].

Other studies reported much lower pneumonia CFRs, including two studies from Afghanistan that reported CFRs of 4.3% among children U5 admitted to a regional hospital in Balkh province [24], and 0.7% among children aged under 36 months enrolled in an RCT in the capital Kabul (with no data on deaths prior to study enrolment) [35]. Data from MSF hospitals in Somalia and Syria showed a pneumonia CFR among children and adolescents of 2.1% and 0.5% (0.7% for U5 population) respectively [23,28]. Birindwa et al’s before-after study reported a pneumonia CFR of 4.8% among children U5 hospitalised in DRC, with no change after the introduction of PCV-13 (OR 1.06, 95% CI = 0.52-2.13, *P* = 0.86) [27].

Three of these studies reported on risk factors for death, adjusting for a variety of individual and social covariates, finding that mortality was associated with lower household socioeconomic status (eg, maternal education, house structure), malnutrition and other chronic conditions [22,26,27].

### Other key findings

Few studies reported data on household, population or contextual risk (or protective) factors for pneumonia, or pneumonia death (Table S3 in the **Online Supplemental Document**, results reported above).

Three studies reported data on pneumonia aetiology based on testing of nasopharyngeal samples for *Streptococcus pneumoniae* or viruses (Table S3 in the Online Supplemental Document) [15,25,36]. In a regional hospital of Afghanistan, researchers detected *S. pneumoniae* in nasopharyngeal samples of 38% (24/326) of children admitted with pneumonia. However, half of participants were not swabbed due to severe disease, so this represents the less unwell of this cohort. Multiple studies by a research group working in Thai refugee camps, a context without PCV or Hib vaccine, found that viruses were detected in the majority of nasopharyngeal samples taken from children with pneumonia [15,25]. Detection rates were similar among children recruited from the community and those presenting to facilities (61.3% and 53.7% respectively) with respiratory syncytial virus (RSV) detected most commonly (33.9% and 24.9%). Multiple viruses were more commonly detected in the community cohort (17.6% vs 4.0%) [15,25].

Studies generally reported minimal data on contextual factors related to pneumonia protection, prevention, or treatment making it difficult to meaningfully compare across settings (Table S3 in the **Online Supplemental Document**).

## DISCUSSION

In this review, we found that pneumonia was a leading infectious cause of morbidity and mortality in children and adolescents affected by humanitarian emergencies in LMICs, with the greatest burden found in children U5. We found pneumonia is particularly common in camp settings, and in newly arrived populations. We found significant heterogeneity between contexts, and a lack of uniformity in reporting morbidity and mortality data.

We identified pneumonia incidence data from four papers involving 19 countries; most from refugee camps. Pneumonia incidence ranged between 71 and 146 cases per 100 person-years, higher than findings in the 2010 Bellos review that only included data from Iran and Nicaragua (31 and 110 per 100 person-years). We identified one notable outlier involving two refugee camps in Cox’s Bazar, Bangladesh, where household survey data suggested that 52% of children had pneumonia in the past 2 weeks (and 23% had diarrhoea) [14]. This data could be spurious, representing the challenges of household surveys among a population who may be incentivised to report higher needs in order to access survival essentials. However, this was during acute displacement, just 2-3 months after the displacement of half a million Rohingya to the bordering district of Cox’s Bazar, and two-thirds of those with reported pneumonia or diarrhoea also reported seeking care at a formal health facility – suggesting they had real health concerns [14]. Data from the Bangladesh Demographic & Household Survey 2017-18 show that 3% of children under 5 had symptoms of ARI in the past 2 weeks, 40% of whom were taken to a formal health facility [37]. Keeping in mind limitations of this data collection method, this comparison suggests refugee children in Cox’s Bazar were more than 17 times more likely to have had symptoms consistent with ARI in the last fortnight.

We found that pneumonia was the primary cause for around 15%-20% of child illnesses in all types of humanitarian settings, common in both community/outpatient (10%-34%) and inpatient settings (7%-58%) and accounted for around 20% of deaths. These estimates were generally higher than the proportional morbidity reported in the 2010 Bellos review (3%-9% of community/outpatient cases, 4%-34% of inpatient cases, 6%-40% of deaths). We identified one study which found that introduction of a pneumonia vaccine (PCV-13) in a complex humanitarian conflict setting reduced proportional morbidity from pneumonia [27].

The variability in morbidity and mortality estimates we found is not unexpected, given the diversity and complexity of humanitarian emergencies and the populations affected. We know that populations affected vary considerably in terms of baseline health status, demographics, access to health care, and health system resilience, and that different disasters will impact populations in different ways and over different periods of time. Indeed, this diversity and complexity highlights the need for contextually-appropriate recommendations and interventions informed by local data.

Overall, it is clear that pneumonia is a key contributor to increased infectious disease burden and mortality in humanitarian settings, for younger and older children alike [2,3,5].Pneumonia should be a priority concern for humanitarian programming and emergency contexts should be a priority concern for child pneumonia programming – including older children. We should consider extending pneumonia interventions, such as pneumococcal vaccine, to older children in emergency contexts, particularly where undernutrition is prevalent.

### Comparison with non-crisis settings

We found few studies that compared crisis- and non-crisis-affected populations. These suggested an increase in pneumonia morbidity in the acute phase following natural disasters [29,31,32], but no clear relationship between hospital-based pneumonia morbidity or mortality and crisis acuity in conflict settings [18,22,23]. This is likely due to a complex interplay between population displacement, changes in care-seeking behaviour and living environments, and access to health services. However, more and better studies are required to better understand the relationships between crisis, populations, context, and pneumonia outcomes.

Further comparisons between crisis-affected and non-crisis-affected populations are challenging and rely on broad regional and global disease burden estimates. Our review covered a 21-year period, during which global child mortality has decreased substantially. The United Nations 2020 report on child mortality found that the U5 mortality rate decreased by 50% from 2000 to 2019, from 76 to 38 deaths per 1000 livebirths [37]. However, progress has not been uniform, with countries classified as ‘fragile’ recording under-five mortality rates three times higher than other countries and most are at risk of missing the SDG target for under-five mortality by 2030 [37].

Estimates suggest that pneumonia incidence decreased by 30% in LMICs between 2000 and 2015, from 33 to 23 episodes per 100 child-years, U5 deaths from pneumonia decreased from 1.7 million to 0.9 million (from 15 to 7 deaths per 1000 livebirths), and CFRs for pneumonia and severe pneumonia decreased from 0.96% to 0.65% and 6.1% to 4.2%, respectively [37]. The incidence rates found in our review suggest that children affected by humanitarian emergencies are at 3 to 10 times higher risk of pneumonia than children in LMICs in general – and that this risk is even higher for those recently displaced or living in crowded, unsanitary conditions [37]. Similarly, limited data on population-level pneumonia mortality incidence (0.5 to 4.2 deaths per 1000 child-years) and hospital-based case fatality rates suggest higher risk of death from pneumonia in humanitarian settings, with considerable variation between heterogeneous humanitarian settings.

### Limitations

The 2010 review by Bellos et al. recommended better pneumonia surveillance, use of clear case definitions, disaggregation of data by age, and population-level morbidity and mortality [5]. While we found some important recent contributions to the literature, most of Bellos et al.’s concerns about data quality, heterogeneity of measurement and reporting, and lack of detail on child pneumonia remain. Case definitions for pneumonia and data reporting methods were variable, denominator populations were often poorly defined, few studies reported morbidity or mortality at a population level, and most studies did not stratify results according to age. This limited our ability to draw meaningful comparisons between populations, contexts, or age groups, or perform any quantitative synthesis.

As a scoping review, we used broad inclusion criteria to ensure wide capture of relevant studies. However, we had to exclude many studies that should have provided valuable data due to unclear case definitions for pneumonia or incomplete reporting of morbidity and mortality data. To ensure we captured programmatic grey literature we contacted individuals and networks working in humanitarian child health. This revealed few additional studies but did highlight deficiencies and opportunities in data collection and reporting of child health in humanitarian emergencies – particularly regarding linkage of facility-level data to populations served.

Our findings include considerable variability between settings, with a high degree of heterogeneity between populations, reporting methods, and outcome measures. We note that facility-based reports of pneumonia incidence are likely to underestimate true incidence, due to challenges with care-seeking and service access, and case ascertainment. Furthermore, facility-based mortality estimates often did not include deaths prior to admission (ie, deaths in the community before presentation to hospital), and this may artificially deflate case fatality and mortality incidence rates.

## CONCLUSIONS

Pneumonia is a leading cause of morbidity and mortality in children and adolescents affected by humanitarian emergencies. Our review found a higher burden of disease than has previously been reported, spanning all geographies and types of emergencies and with variation between specific contexts. This warrants greater emphasis on child pneumonia in humanitarian programming and inclusion of older children as a priority target. Future research should address population pneumonia burden, particularly for older children and adolescents, and describe contextual factors to allow for more meaningful interpretation and guide future programmatic and health responses.

## Additional material


Online Supplementary Document

## References

[1] Vos T Lim SS Abbafati C Abbas KM Abbasi M Abbasifard M Global burden of 369 diseases and injuries in 204 countries and territories, 1990-2019: a systematic analysis for the Global Burden of Disease Study 2019. Lancet. 2020;396:1204-22. 10.1016/S0140-6736(20)30925-9 33069326 PMC7567026

[2] Connolly MA Gayer M Ryan MJ Salama P Spiegel P Heymann DL Communicable diseases in complex emergencies: impact and challenges. Lancet. 2004;364:1974-83. 10.1016/S0140-6736(04)17481-3 15567014

[3] Checchi F, Gayer M, Grais R, Mills EJ. Public health in crisis-affected populations. A practical guide for decision-makers. 2007.

[4] WHO/UNICEF. Ending Preventable Child Deaths from Pneumonia and Diarrhoea by 2025: The integrated Global Action Plan for Pneumonia and Diarrhoea (GAPPD). Geneva: WHO; 2013. 10.1136/archdischild-2013-305429 25613963

[5] Bellos A Mulholland K O’Brien KL Qazi SA Gayer M Checchi F The burden of acute respiratory infections in crisis-affected populations: a systematic review. Confl Health. 2010;4:3. 10.1186/1752-1505-4-3 20181220 PMC2829474

[6] Moher D Shamseer L Clarke M Ghersi D Liberati A Petticrew M Preferred reporting items for systematic review and meta-analysis protocols (PRISMA-P) 2015 statement. Syst Rev. 2015;4:1-9. 10.1186/2046-4053-4-1 25554246 PMC4320440

[7] Tricco AC Lillie E Zarin W O’Brien KK Colquhoun H Levac D PRISMA Extension for Scoping Reviews (PRISMA-ScR): Checklist and Explanation. Ann Intern Med. 2018;169:467-73. 10.7326/M18-0850 30178033

[8] Thomas BH Ciliska D Dobbins M Micucci S A Process for Systematically Reviewing the Literature: Providing the Research Evidence for Public Health Nursing Interventions. Worldviews Evid Based Nurs. 2004;1:176-84. 10.1111/j.1524-475X.2004.04006.x 17163895

[9] Armijo-Olivo S Stiles CR Na H Biondo PD Cummings GG Assessment of study quality for systematic reviews: A comparison of the Cochrane Collaboration Risk of Bias Tool and the Effective Public Health Practice Project Quality Assessment Tool: Methodological research. J Eval Clin Pract. 2012;18:12-8. 10.1111/j.1365-2753.2010.01516.x 20698919

[10] Farrah K Young K Tunis MC Zhao L Risk of bias tools in systematic reviews of health interventions: an analysis of PROSPERO-registered protocols. Syst Rev. 2019;8:280. 10.1186/s13643-019-1172-8 31730014 PMC6857304

[11] Arksey H O’Malley L Scoping studies: towards a methodological framework. Int J Soc Res Methodol. 2005;8:19-32. 10.1080/1364557032000119616

[12] Peters MD Godfrey CM Khalil H McInerney P Parker D Soares CB Guidance for conducting systematic scoping reviews. Int J Evid-Based Healthc. 2015;13:141-6. 10.1097/XEB.0000000000000050 26134548

[13] Sucharew H Macaluso M Methods for research evidence synthesis: the scoping review approach. J Hosp Med. 2019;14:416-8. 10.12788/jhm.3248 31251164

[14] Summers A Humphreys A Leidman E Van Mil LT Wilkinson C Narayan A Notes from the Field: Diarrhea and Acute Respiratory Infection, Oral Cholera Vaccination Coverage, and Care-Seeking Behaviors of Rohingya Refugees - Cox’s Bazar, Bangladesh, October-November 2017. MMWR Morb Mortal Wkly Rep. 2018;67:533-5. 10.15585/mmwr.mm6718a6 29746454 PMC5944978

[15] Turner C Turner P Carrara V Burgoine K Tha Ler Htoo S Watthanaworawit W High rates of pneumonia in children under two years of age in a South East Asian refugee population. PLoS One. 2013;8:e54026. 10.1371/journal.pone.0054026 23320118 PMC3539989

[16] Manaseki-Holland S Maroof Z Bruce J Mughal MZ Masher MI Bhutta ZA Effect on the incidence of pneumonia of vitamin D supplementation by quarterly bolus dose to infants in Kabul: a randomised controlled superiority trial. Lancet. 2012;379:1419-27. 10.1016/S0140-6736(11)61650-4 22494826 PMC3348565

[17] Hershey CL Doocy S Anderson J Haskew C Spiegel P Moss WJ Incidence and risk factors for Malaria, pneumonia and diarrhea in children under 5 in UNHCR refugee camps: A retrospective study. Confl Health. 2011;5:24. 10.1186/1752-1505-5-24 22029694 PMC3223490

[18] van Berlaer G Elsafti AM Al Safadi M Souhil Saeed S Buyl R Debacker M Diagnoses, infections and injuries in Northern Syrian children during the civil war: A cross-sectional study. PLoS One. 2017;12:e0182770. 10.1371/journal.pone.0182770 28886038 PMC5590741

[19] Anwar MY Burnham G Trends in infectious disease incidence among children in Afghanistan at a time of public health services expansion. East Mediterr Health J. 2017;22:778-85. 10.26719/2016.22.11.778 28177107

[20] Clarke-Deelder E Shapira G Samaha H Fritsche GB Fink G Quality of care for children with severe disease in the Democratic Republic of the Congo. BMC Public Health. 2019;19:1608. 10.1186/s12889-019-7853-3 31791291 PMC6889659

[21] Bernasconi A Crabbé F Rossi R Qani I Vanobberghen A Raab M The ALMANACH Project: Preliminary results and potentiality from Afghanistan. Int J Med Inform. 2018;114:130-5. 10.1016/j.ijmedinf.2017.12.021 29330009

[22] Sodemann M Veirum J Biai S Nielsen J Bale C Skytte Jakobsen M Reduced case fatality among hospitalized children during a war in Guinea-Bissau: a lesson in equity. Acta Paediatr. 2004;93:959-64. 10.1111/j.1651-2227.2004.tb02696.x 15303813

[23] Ngoy BB Zachariah R Hinderaker SG Khogali M Manzi M van Griensven J Paediatric in-patient care in a conflict-torn region of Somalia: are hospital outcomes of acceptable quality? Public Health Action. 2013;3:125-7. 10.5588/pha.12.0104 26393014 PMC4463110

[24] Rasooly MH Shirpoor A Epidemiology and associated factors of severity of pneumonia among children under five in north Afghanistan in 2018: Experiences from a chronic conflict setting. Oman Med J. 2020;35:13.

[25] Turner P Turner C Watthanaworawit W Carrara V Cicelia N Deglise C Respiratory virus surveillance in hospitalised pneumonia patients on the Thailand-Myanmar border. BMC Infect Dis. 2013;13:434. 10.1186/1471-2334-13-434 24498873 PMC3847692

[26] Zabihullah R Dhoubhadel BG Rauf FA Shafiq SA Suzuki M Watanabe K Risk for Death among Children with Pneumonia, Afghanistan. Emerg Infect Dis. 2017;23:1404-8. 10.3201/eid2308.151550 28726625 PMC5547795

[27] Birindwa AM Manegabe JT Mindja A Nordén R Andersson R Skovbjerg S Decreased number of hospitalized children with severe acute lower respiratory infection after introduction of the pneumococcal conjugate vaccine in the Eastern Democratic Republic of the Congo. Pan Afr Med J. 2020;37:211. 10.11604/pamj.2020.37.211.22589 33520050 PMC7821803

[28] Meiqari L Hoetjes M Baxter L Lenglet A Impact of war on child health in northern Syria: the experience of Medecins Sans Frontieres. Eur J Pediatr. 2018;177:371-80. 10.1007/s00431-017-3057-y 29255951 PMC5816770

[29] van Berlaer G de Jong F Das T Gundran CP Samyn M Gijs G Clinical Characteristics of the 2013 Haiyan Typhoon Victims Presenting to the Belgian First Aid and Support Team. Disaster Med Public Health Prep. 2019;13:265-78. 10.1017/dmp.2018.54 29970208

[30] Huerga H Vasset B Prados E Adult and paediatric mortality patterns in a referral hospital in Liberia 1 year after the end of the war. Trans R Soc Trop Med Hyg. 2009;103:476-84. 10.1016/j.trstmh.2008.12.004 19243803

[31] Chang MP Simkin DJ de Lara ML Kirsch TD Characterizing Hospital Admissions to a Tertiary Care Hospital After Typhoon Haiyan. Disaster Med Public Health Prep. 2016;10:240-7. 10.1017/dmp.2015.165 26832860

[32] Giri BR Chapagain RH Sharma S Shrestha S Ghimire S Shankar PR Effect of the 2015 earthquake on pediatric inpatient pattern at a tertiary care hospital in Nepal. BMC Pediatr. 2018;18:28. 10.1186/s12887-018-1008-z 29402263 PMC5800012

[33] World Bank. Child and Infant Mortality Rate data 2020. Washington DC: World Bank; 2020.

[34] Robinson E Lee L Roberts LF Poelhekke A Charles X Ouabo A Mortality beyond emergency threshold in a silent crisis–results from a population-based mortality survey in Ouaka prefecture, Central African Republic, 2020. Confl Health. 2021;15:50. 10.1186/s13031-021-00385-2 34193238 PMC8243074

[35] Manaseki-Holland S Qader G Masher MIM Bruce J Mughal MZ Chandramohan D Effects of vitamin D supplementation to children diagnosed with pneumonia in Kabul: A randomised controlled trial. Trop Med Int Health. 2010;15:1148-55. 10.1136/adc.2010.186338.138 20723187

[36] Turner C Turner P Cararra V Eh Lwe N Watthanaworawit W Day NP A high burden of respiratory syncytial virus associated pneumonia in children less than two years of age in a South East Asian refugee population. PLoS One. 2012;7:e50100. 10.1371/journal.pone.0050100 23185545 PMC3502361

[37] National Institute of Population Research and Training (NIPORT), and ICF. Bangladesh Demographic and Health Survey 2017-18. Dhaka, Bangladesh, and Rockville, Maryland, USA: NIPORT and ICF; 2020.

[38] Estimation UNICEF. Levels & Trends in Child Mortality: Report 2019. New York, NY: UNICEF, WHO, World Bank; 2019.

