# Supplementary material for: Childhood pneumonia in humanitarian emergencies in low- and middle-income countries: A systematic scoping review
Source: J Glob Health. 2022 Apr 9;12:10001. doi: 10.7189/jogh.12.10001 (PMC8980764; doi:10.7189/jogh.12.10001)
Supplement: Online Supplementary Document [file jogh-12-10001-s001.pdf]

Supplemental material

Chen SJ, Walker PJB, Mulholland K, Graham HR; ARI Review group. Childhood pneumonia in humanitarian emergencies in low- and middle-income countries: A systematic scoping review. J Glob Health 2022

Table of contents

Text S1. Additional detail on Methods 2
Text S2. MEDLINE (Ovid) search strategy 4
Text S3. Effective Public Health Practice Project (EPHPP) Quality Assessment Tool For Quantitative Studies Component Ratings 6
Figure S1. Geographical distribution of reported data in included studies (n = 22) 14
Table S1. Pneumonia incidence and proportional morbidity among children in humanitarian emergencies in low- and middle-income countries. 15
Table S2. Proportional mortality, case fatality rates, and incidence of death from pneumonia among children in humanitarian emergencies in low- and middle-income countries. 17
Table S3. Other key findings from studies included in this review. 19

Text S1. Additional detail on Methods

Protocol and registration
We drafted the protocol for this review using the Preferred Reporting Items for Systematic Reviews and Meta-analysis Protocols (PRISMA-P) extension for scoping reviews guidelines. PROSPERO does not accept scoping review protocols for registration.

Inclusion and exclusion criteria
We included all studies that presented quantitative data on pneumonia and/or severe pneumonia as defined by WHO (with burden indicators such as incidence or prevalence of disease, proportional morbidity, proportional mortality and/or case-fatality rate) in children and adolescents aged 1 month to 18 years old affected by a humanitarian emergency (e.g. natural disasters, forced displacement, refugee camps, famines, and armed conflict/war) in LMICs. Country income level was determined using the World Bank classification at the time of data collection in a given study. We also included studies that diagnosed pneumonia radiologically, microbiologically, or via viral nucleic acid testing.

Where possible, we included studies that:

I. Compared the study population to populations not affected by a humanitarian emergency (e.g. a comparable area not affected by a humanitarian emergency, or the same location before, and/or after the occurrence of a humanitarian emergency); and

II. Presented original data on risk factors and aetiology of WHO-defined pneumonia (or severe pneumonia), pneumonia prevention programmes (e.g. vaccination, improvements in water, sanitation, and household air pollution) and/or pneumonia treatment programmes (e.g. case management, antibiotics, and oxygen therapy).

Peer-reviewed journal articles were eligible for inclusion if they were published in English from 2000–2020. We included interventional studies, observational studies, descriptive studies, large case series with >50 participants, conference abstracts (where reported data was not available elsewhere), and meta-analyses.

We excluded studies which:

I. Grouped together upper and lower respiratory tract infections, or did not use a clear definition of pneumonia;

II. Focused on specific respiratory pathogen epidemics (e.g. COVID-19, SARS, MERS) and not to humanitarian emergency contexts more broadly;

III. Did not report child-specific outcomes;

IV. Studied asylum seekers, refugees, or IDPs resettled in high-income countries after fleeing a humanitarian emergency in a low- or middle- income country; or

V. Centred on health promotion programmes not specifically related to pneumonia.

We also excluded case reports, case series with <50 participants, review articles, and editorial or commentary articles. Where possible, we disaggregated outcome data by age (infants 1-12 months, children 1-4 years, older children 5-9 years, and adolescents 10-18 years), sex, and disability, and compared variation in outcomes between different settings.

Information sources and search strategy
We searched the following MEDLINE, EMBASE, and PubMed for peer-reviewed articles in July 2020, excluding opinion, commentary, editorial and review papers that do not present original data or analysis. We used search terms relating to “children/adolescents”, “pneumonia/lower respiratory tract infection”, and “humanitarian/emergency/refugee”, using synonyms and mapping search terms to subject headings where appropriate, to retrieve all relevant articles. Exact search terms can be found in Appendix 2. An experienced research librarian applied a custom-built search filter to our search strategy to limit results to low- and middle-income countries, and to exclude publications relating to respiratory pathogen epidemics (such as Covid-19, SARS, and MERS) that did not relate to humanitarian emergencies more broadly.

To supplement our electronic database search, we also searched grey literature for non-peer-reviewed articles, including reports from UN agencies (e.g. WHO, UNICEF, UNHCR) and other independent organisations (e.g. Sphere, MSF) through key website searches and contact with organisation staff; scanned reference lists of included articles and related systematic reviews for relevant reports; and contacted authors of potentially relevant studies to request unpublished results.

Study selection
Two investigators (SC and PW) independently screened the titles and abstracts of all unique citations against our predetermined inclusion and exclusion criteria, then reviewed full-text articles for inclusion. We resolved discrepancies at any stage by discussion with final arbitration by a third investigator (HG).

Data extraction and management

Two investigators (SC and PW) jointly developed a data extraction form to extract all data relevant to our review; one investigator then systematically extracted relevant data from each included study onto this template which was checked for accuracy by a third investigator (HG). We extracted the following key variables: type of study design, data collection period, acute respiratory infection or pneumonia case definition, child-specific age groups within study population, type of humanitarian emergency (e.g. natural disaster, forced displacement/refugee camps, armed conflict), healthcare setting (e.g. outpatient clinics), study location (e.g. country, WHO region), indicators of burden for pneumonia and/or severe pneumonia (e.g. number of cases, denominator at risk, proportional morbidity, proportional mortality, case-fatality rate), factors associated with disease or death, and data relating to protection, prevention and/or treatment strategies for pneumonia. Where possible, we extracted data stratified by age. Any disagreements were initially resolved by discussion, with final arbitration by a third investigator (HG).

Synthesis of results

In accordance with scoping review methodology**,** (11-13) we mapped the existing literature using numerical summaries of included studies then described key findings and gaps through narrative synthesis. We did not perform quantitative meta-analysis due to the high degree of heterogeneity in study design, pneumonia case definitions, and outcome measures reported. Instead, we provided summary data for each included study and presented descriptive tables of key findings, including variation between settings. Where possible, we stratified relevant data and findings by age (infants 1-12 months, children 1-5 years, older children 5-9 years, and adolescents 10-19 years) and by severity of pneumonia (pneumonia and severe pneumonia as defined by WHO).

Text S2. MEDLINE (Ovid) search strategy

1. exp *Pneumonia/
2. *Pneumococcal Infections/
3. ((respiratory adj3 infection*) or pneumonia or pneumonias or lung-inflammation* or lobitis or nonspecific-inflammatory-lung-disease* or peripneumonia or pleuropneumonia or pleuropneumonitis or pneumonic-lung* or pneumonic-pleurisy or pneumonic-pleuritis or pneumonitides or pneumonitis or pulmonal-inflammation* or pulmonary-inflammation* or pulmonic-inflammation*).tw,kf.
4. *Bacteremia/
5. exp *empyema, pleural/ or exp *pleural effusion/
6. (bacter?emia or empyema or pleural or bronchial or bronchoalveolar or alveolar or endotracheal or tracheal).tw,kf.
7. *Streptococcus pneumoniae/
8. exp *Haemophilus/
9. (pneumoniae or pneumococc* or haemophilus).tw,kf,hw.
10. (4 or 5 or 6) and (7 or 8 or 9)
11. exp *Bronchiolitis/
12. bronchiolitis.tw,kf.
13. 1 or 2 or 3 or 10 or 11 or 12
14. exp *natural disasters/
15. (natural-disaster? or avalance? or storm? or cyclone? or drought? or earthquake? or famine? or flood? or catastrophic-flooding? or landslide? or land-slide? or mudslide? or mud-slide? or rockslide? or rock-slide? or tidal-wave? or tidalwave? or earth-tide? or earthtide? or ocean-tide? or oceantide? or tsunami? or hurricane? or tornado* or typhoon? or wildfire? or bushfire or brushfire? or fire? or volcano* or volcanic).tw,kf.
16. *"warfare and armed conflicts"/ or exp *armed conflicts/
17. afghanistan/ or yemen/ or Syria/ or central african republic/ or "democratic republic of the congo"/ or ethiopia/ or somalia/ or south sudan/ or Venezuela/
18. *Refugees/ or *Refugee Camps/
19. (Refugee* or crisis-affected-population* or armed-conflict* or complex-emergenc* or displaced or forced-displacement* or forced-migration* or humanitarian or war or wars or yemen or syria or (democratic-republic adj3 congo) or south-sudan or afghanistan or central-african-republic or venezuela or ethiopia or darfur or somalia or rohingya).tw,kf.
20. *disease outbreaks/ or *epidemics/ or *pandemics/
21. *west nile virus/ or *west nile fever/
22. *Influenza A Virus, H1N1 Subtype/ or *severe acute respiratory syndrome/
23. *Hemorrhagic Fever, Ebola/
24. *Zika Virus Infection/ or *Zika Virus/
25. *Poliovirus/
26. *Ebolavirus/
27. *middle east respiratory syndrome coronavirus/ or *sars virus/
28. (H1N1* or novel-influenza-A or swineflu or swine-flu or 2009-flu or 2009-influenza or polio* or ebola* or EVD or zika* or MERS or middle-east-respiratory-syndrome or MERS-CoV or SARS or west-nile-virus or west-nile-fever or wnv).tw,kf.
29. (PHEIC? or epidemic? or pandemic? or disease-outbreak? or disease-out-break? or virus-outbreak? or virus-out-break? or public-health-emergency-of-international-concern).tw,kf.
30. 14 or 15 or 16 or 17 or 18 or 19 or 20 or 21 or 22 or 23 or 24 or 25 or 26 or 27 or 28 or 29
31. *morbidity/ or *incidence/ or *prevalence/ or exp *mortality/
32. *risk/ or *risk assessment/ or *risk factors/
33. *"cost of illness"/
34. *treatment outcome/
35. *public health/ or *epidemiology/ or *pharmacoepidemiology/ or *preventive medicine/
36. (pnu-im?une or pnuim?une or pcv10 or pcv-10 or pcv13 or pcv-13 or prevenar13 or prevenar-13 or prevnar13 or prevnar-13).tw,kf.
37. ((10-valent or ten-valent or 13-valent or thirteen-valent) and (pneumococcal adj5 vaccine*)).tw,kf.
38. ((Hib or Haemophilus-influenzae-type-b or DTP3 or Diphtheria-tetanus-pertussis or measles) and (vaccine* or immuni#ation*)).tw,kf.
39. exp *Pneumococcal Vaccines/ or *Haemophilus Vaccines/ or exp *Measles Vaccine/ or *Diphtheria-Tetanus-Pertussis Vaccine/
40. exp *Malnutrition/
41. exp *HIV Infections/
42. exp *Anti-Bacterial Agents/tu [Therapeutic Use]
43. *Breast Feeding/
44. *hand hygiene/ or *hand disinfection/
45. *air pollution, indoor/

46. *Health Knowledge, Attitudes, Practice/ or *"treatment adherence and compliance"/ or exp *"patient acceptance of health care"/
47. exp *"Referral and Consultation"/
48. *patient care planning/ or *case management/
49. *Managed Care Programs/
50. exp *Respiration, Artificial/
51. *evaluation studies as topic/ or *program evaluation/
52. exp *Mass Screening/
53. (mortalit* or death* or surviv* or case-fatalit* or morbidit* or risk or risks or epidemiolog* or prevent* or outbreak* or prevalence or incidence or disease-burden or burden-of-disease or burden-of-illness or outcome* or public-health or antibiotic* or amoxicillin* or amoxycillin* or gentamicin* or gentamycin* or co-trimoxazole or cotrimoxazole or malnutrition or malnourish* or HIV or breastfeeding or breast-feeding or handwash* or hand-wash* or improved-water or sanitation or air-pollution or (clean adj3 fuel*) or care-seeking or referral* or case-management or ventilation or ventilator* or respiratory-support or mass-screen* or intervention* or evaluation*).tw,kf.
54. 31 or 32 or 33 or 34 or 35 or 36 or 37 or 38 or 39 or 40 or 41 or 42 or 43 or 44 or 45 or 46 or 47 or 48 or 49 or 50 or 51 or 52 or 53
55. (infan* or toddler* or pre-schooler* or preschooler* or kinder or kinders or kindergarten* or kinder-aged or boy or boys or girl or girls or child or children or childhood or pediatric* or paediatric* or school-age* or schoolage* or schoolchild* or schoolgirl* or schoolboy* or adolescen* or youth or youths or teen or teens or teenage*).af.
56. 13 and 30 and 54 and 55
57. limit 56 to (english language and yr="2000 -Current")
58. (((Coronavirus* or corona-virus* or nCov) and ("2019" or wuhan or china or chinese or hubei)) or COV-2 or COV2 or COVID-19 or COVID19 or COVID-2019 or COVID2019 or 2019-nCoV or nCov-2019 or Coronavirus-2 or Coronavirus2 or coronavirus-disease-2019 or corona-virus-disease-2019).tw,kf.
59. Betacoronavirus/ or Coronavirus Infections/
60. limit 57 to covid-19
61. 57 not (58 or 59 or 60)
62. limit 61 to (case reports or comment or editorial or letter)
63. 61 not 62

Text S3. Effective Public Health Practice Project (EPHPP) Quality Assessment Tool For Quantitative Studies Component Ratings

A) SELECTION BIAS

(Q1) Are the individuals selected to participate in the study likely to be representative of the target population?

1 Very likely
2 Somewhat likely
3 Not likely
4 Can't tell

(Q2) What percentage of selected individuals agreed to participate?

1 80 - 100% agreement
2 60 – 79% agreement
3 less than 60% agreement
4 Not applicable
5 Can't tell

RATE THIS SECTION	STRONG	MODERATE	WEAK	
**See dictionary**	1	2	3	Not Applicable

B) STUDY DESIGN

Indicate the study design

1 Randomized controlled trial
2 Controlled clinical trial
3 Cohort analytic (two group pre + post)
4 Case-control
5 Cohort (one group pre + post (before and after))
6 Interrupted time series
7 Other specify ______________________________
8 Can't tell

Was the study described as randomized? If NO, go to Component C.

No Yes

If Yes, was the method of randomization described? (See dictionary)

No Yes

If Yes, was the method appropriate? (See dictionary)

No Yes

RATE THIS SECTION	STRONG	MODERATE	WEAK	
**See dictionary**	1	2	3	Not Applicable

C) # CONFOUNDERS

(Q1) Were there important differences between groups prior to the intervention?

1 Yes
2 No
3 Can't tell

The following are examples of confounders:

1 Race
2 Sex
3 Marital status/family
4 Age
5 SES (income or class)
6 Education
7 Health status
8 Pre-intervention score on outcome measure

(Q2) If yes, indicate the percentage of relevant confounders that were controlled (either in the design (e.g.stratification, matching) or analysis)?

1 80 – 100% (most)
2 60 – 79% (some)
3 Less than 60% (few or none)
4 Can't Tell

RATE THIS SECTION	STRONG	MODERATE	WEAK	
See dictionary	1	2	3	Not Applicable

D) # BLINDING

(Q1) Was (were) the outcome assessor(s) aware of the intervention or exposure status of participants?

1 Yes
2 No
3 Can't tell

(Q2) Were the study participants aware of the research question?

1 Yes
2 No
3 Can't tell

RATE THIS SECTION	STRONG	MODERATE	WEAK	
See dictionary	1	2	3	Not Applicable

E) # DATA COLLECTION METHODS

(Q1) Were data collection tools shown to be valid?

1 Yes
2 No
3 Can't tell

(Q2) Were data collection tools shown to be reliable?

1 Yes
2 No
3 Can't tell

RATE THIS SECTION	STRONG	MODERATE	WEAK	
**See dictionary**	1	2	3	Not Applicable

F) # WITHDRAWALS AND DROP-OUTS

(Q1) Were withdrawals and drop-outs reported in terms of numbers and/or reasons per group?

1 Yes
2 No
3 Can't tell
4 Not Applicable (i.e. one time surveys or interviews)

(Q2) Indicate the percentage of participants completing the study. (If the percentage differs by groups, record thelowest).

1 80 -100%
2 60 - 79%
3 less than 60%
4 Can't tell
5 Not Applicable (i.e. Retrospective case-control)

RATE THIS SECTION	STRONG	MODERATE	WEAK	
**See dictionary**	1	2	3	Not Applicable

G) # INTERVENTION INTEGRITY

(Q1) What percentage of participants received the allocated intervention or exposure of interest?

1 80 -100%
2 60 - 79%
3 less than 60%
4 Can't tell

(Q2) Was the consistency of the intervention measured?

1 Yes
2 No
3 Can't tell

(Q3) Is it likely that subjects received an unintended intervention (contamination or co-intervention) that mayinfluence the results?

4 Yes
5 No
6 Can't tell

H) # ANALYSES

(Q1) Indicate the unit of allocation (circle one)

community organization/institution practice/office individual

(Q2) Indicate the unit of analysis (circle one)

community organization/institution practice/office individual

(Q3) Are the statistical methods appropriate for the study design?

1 Yes
2 No
3 Can't tell

(Q4) Is the analysis performed by intervention allocation status (i.e. intention to treat) rather than the actual intervention received?

1 Yes
2 No
3 Can't tell

GLOBAL RATING
COMPONENT RATINGS

Please transcribe the information from the gray boxes on pages 1-4 onto this page. See dictionary on how to rate this section.

**A**	**SELECTION BIAS**	**STRONG**	**MODERATE**	**WEAK**	
		1	2	3	
**B**	**STUDY DESIGN**	**STRONG**	**MODERATE**	**WEAK**	
		1	2	3	
**C**	**CONFOUNDERS**	**STRONG**	**MODERATE**	**WEAK**	
		1	2	3	
**D**	**BLINDING**	**STRONG**	**MODERATE**	**WEAK**	
		1	2	3	
**E**	**DATA COLLECTION METHOD**	**STRONG**	**MODERATE**	**WEAK**	
		1	2	3	
**F**	**WITHDRAWALS AND DROPOUTS**	**STRONG**	**MODERATE**	**WEAK**	
		1	2	3	Not Applicable

GLOBAL RATING FOR THIS PAPER (circle one):

1 STRONG (no WEAK ratings)

2 MODERATE (one WEAK rating)

3 WEAK (two or more WEAK ratings)

With both reviewers discussing the ratings:

Is there a discrepancy between the two reviewers with respect to the component (A-F) ratings?
No Yes

If yes, indicate the reason for the discrepancy

1 Oversight
2 Differences in interpretation of criteria
3 Differences in interpretation of study

<u>Final decision of both reviewers (circle one)</u>: **1 STRONG**

2 MODERATE

3	WEAK

EPHPP Quality Assessment Tool For Quantitative Studies Dictionary

The purpose of this dictionary is to describe items in the tool thereby assisting raters to score study quality. Due to under-reporting or lack of clarity in the primary study, raters will need to make judgements about the extent that biasmay be present. When making judgements about each component, raters should form their opinion based upon information contained in the study rather than making inferences about what the authors intended.

A) SELECTION BIAS

(Q1) Participants are more likely to be representative of the target population if they are randomly selected from a comprehensive list of individuals in the target population (score very likely). They may not be representative if they are referred from a source (e.g. clinic) in a systematic manner (score somewhat likely) or self-referred (score not likely).

(Q2) Refers to the % of subjects in the control and intervention groups that agreed to participate in the study before they were assigned to intervention or control groups.

B) STUDY DESIGN

In this section, raters assess the likelihood of bias due to the allocation process in an experimental study. For observational studies, raters assess the extent that assessments of exposure and outcome are likely to be independent. Generally, the type of design is a good indicator of the extent of bias. In stronger designs, an equivalent control group is present and the allocation process is such that the investigators are unable to predict the sequence.

Randomized Controlled Trial (RCT)

An experimental design where investigators randomly allocate eligible people to an intervention or control group. A rater should describe a study as an RCT if the randomization sequence allows each study participant to have the same chance of receiving each intervention and the investigators could not predict which intervention was next. If the investigators do not describe the allocation process and only use the words 'random' or 'randomly', the study is described as a controlled clinical trial.

See below for more details.

Was the study described as randomized?

Score YES, if the authors used words such as random allocation, randomly assigned, and random assignment.

Score NO, if no mention of randomization is made.

Was the method of randomization described?

Score YES, if the authors describe any method used to generate a random allocation sequence.

Score NO, if the authors do not describe the allocation method or describe methods of allocation such as alternation, case record numbers, dates of birth, day of the week, and any allocation procedure that is entirely transparent before assignment, such as an open list of random numbers of assignments.
If NO is scored, then the study is a controlled clinical trial.

Was the method appropriate?

Score YES, if the randomization sequence allowed each study participant to have the same chance of receiving each intervention and the investigators could not predict which intervention was next. Examples of appropriate approaches include assignment of subjects by a central office unaware of subject characteristics, or sequentially numbered, sealed, opaque envelopes.

Score NO, if the randomization sequence is open to the individuals responsible for recruiting and allocating participants or providing the intervention, since those individuals can influence the allocation process, either knowingly or unknowingly.

If NO is scored, then the study is a controlled clinical trial.

Controlled Clinical Trial (CCT)

An experimental study design where the method of allocating study subjects to intervention or control groups is open to individuals responsible for recruiting subjects or providing the intervention. The method of allocation is transparent before assignment, e.g. an open list of random numbers or allocation by date of birth, etc.

Cohort analytic (two group pre and post)

An observational study design where groups are assembled according to whether or not exposure to the intervention has occurred. Exposure to the intervention is not under the control of the investigators. Study groups might be non-equivalent or not comparable on some feature that affects outcome.

Case control study

A retrospective study design where the investigators gather 'cases' of people who already have the outcome of interest and 'controls' who do not. Both groups are then questioned or their records examined about whether they received the intervention exposure of interest.

Cohort (one group pre + post (before and after)

The same group is pretested, given an intervention, and tested immediately after the intervention. The intervention group, by means of the pretest, act as their own control group.

Interrupted time series

A time series consists of multiple observations over time. Observations can be on the same units (e.g. individuals over time) or on different but similar units (e.g. student achievement scores for particular grade and school). Interrupted time series analysis requires knowing the specific point in the series when an intervention occurred.

C) CONFOUNDERS

By definition, a confounder is a variable that is associated with the intervention or exposure and causally related to the outcome of interest. Even in a robust study design, groups may not be balanced with respect to important variables prior to the intervention. The authors should indicate if confounders were controlled in the design (by stratification or matching) or in the analysis. If the allocation to intervention and control groups is randomized, the authors must report that the groups were balanced at baseline with respect to confounders (either in the text or a table).

D) BLINDING

(Q1) Assessors should be described as blinded to which participants were in the control and intervention groups. The purpose of blinding the outcome assessors (who might also be the care providers) is to protect against detection bias.

(Q2) Study participants should not be aware of (i.e. blinded to) the research question. The purpose of blinding the participants is to protect against reporting bias.

E) DATA COLLECTION METHODS

Tools for primary outcome measures must be described as reliable and valid. If ‘face’ validity or ‘content’ validity has been demonstrated, this is acceptable. Some sources from which data may be collected are described below:

Self reported data includes data that is collected from participants in the study (e.g. completing a questionnaire, survey, answering questions during an interview, etc.).

Assessment/Screening includes objective data that is retrieved by the researchers. (e.g. observations by investigators).

Medical Records/Vital Statistics refers to the types of formal records used for the extraction of the data.

Reliability and validity can be reported in the study or in a separate study. For example, somestandard assessment tools have known reliability and validity.

F) WITHDRAWALS AND DROP-OUTS

Score YES if the authors describe BOTH the numbers and reasons for withdrawals and drop-outs.

Score NO if either the numbers or reasons for withdrawals and drop-outs are not reported.

The percentage of participants completing the study refers to the % of subjects remaining in the study at the final data collection period in all groups (i.e. control and intervention groups).

G) INTERVENTION INTEGRITY

The number of participants receiving the intended intervention should be noted (consider both frequency and intensity). For example, the authors may have reported that at least 80 percent of the participants received the complete intervention. The authors should describe a method of measuring if the intervention was provided to all participants the same way. As well, the authors should indicate if subjects received an unintended intervention that may have influenced the outcomes. For example, co-intervention occurs when the study group receives an additional intervention (other than that intended). In this case, it is possible that the effect of the intervention may be over-estimated.
Contamination refers to situations where the control group accidentally receives the study intervention. This could result in an under-estimation of the impact of the intervention.

H) ANALYSIS APPROPRIATE TO QUESTION

Was the quantitative analysis appropriate to the research question being asked?

An intention-to-treat analysis is one in which all the participants in a trial are analyzed according to the intervention to which they were allocated, whether they received it or not. Intention-to-treat analyses are favoured in assessments of effectiveness as they mirror the noncompliance and treatment changes that are likely to occur when the intervention is used in practice, and because of the risk of attrition bias when participants are excluded from the analysis.

Component Ratings of Study: For each of the six components A – F, use the following descriptions as a roadmap.

A) SELECTION BIAS

Strong: The selected individuals are very likely to be representative of the target population (Q1 is 1) and there is greater than 80% participation (Q2 is 1).

Moderate: The selected individuals are at least somewhat likely to be representative of the target population (Q1 is 1 or 2); and there is 60 - 79% participation (Q2 is 2). 'Moderate' may also be assigned if Q1 is 1 or 2 and Q2 is 5 (can't tell).

Weak: The selected individuals are not likely to be representative of the target population (Q1 is 3); or there is less than 60% participation (Q2 is 3) or selection is not described (Q1 is 4); and the level of participation is not described (Q2 is 5).

B) DESIGN

Strong: will be assigned to those articles that described RCTs and CCTs.

Moderate: will be assigned to those that described a cohort analytic study, a case control study, a cohort design, or an interrupted time series.

Weak: will be assigned to those that used any other method or did not state the method used.

C) CONFOUNDERS

Strong: will be assigned to those articles that controlled for at least 80% of relevant confounders (Q1 is 2); or (Q2 is 1).

Moderate: will be given to those studies that controlled for 60 – 79% of relevant confounders (Q1 is 1) and (Q2 is 2). Weak: will be assigned when less than 60% of relevant confounders were controlled (Q1 is 1) and (Q2 is 3) or control of confounders was not described (Q1 is 3) and (Q2 is 4).

D) BLINDING

Strong: The outcome assessor is not aware of the intervention status of participants (Q1 is 2); and the study participants are not aware of the research question (Q2 is 2).

Moderate: The outcome assessor is not aware of the intervention status of participants (Q1 is 2); or the study participants are not aware of the research question (Q2 is 2); or blinding is not described (Q1 is 3 and Q2 is 3).

Weak: The outcome assessor is aware of the intervention status of participants (Q1 is 1); and the study participants are aware of the research question (Q2 is 1).

E) DATA COLLECTION METHODS

Strong: The data collection tools have been shown to be valid (Q1 is 1); and the data collection tools have been shown to be reliable (Q2 is 1).

Moderate: The data collection tools have been shown to be valid (Q1 is 1); and the data collection tools have not been shown to be reliable (Q2 is 2) or reliability is not described (Q2 is 3).

Weak: The data collection tools have not been shown to be valid (Q1 is 2) or both reliability and validity are not described (Q1 is 3 and Q2 is 3).

F) WITHDRAWALS AND DROP-OUTS - a rating of:

Strong: will be assigned when the follow-up rate is 80% or greater (Q2 is 1).

Moderate: will be assigned when the follow-up rate is 60 – 79% (Q2 is 2) OR Q2 is 5 (N/A).

Weak: will be assigned when a follow-up rate is less than 60% (Q2 is 3) or if the withdrawals and drop-outs were not described (Q2 is 4).

Figure S1. Geographical distribution of reported data in included studies (n = 22)

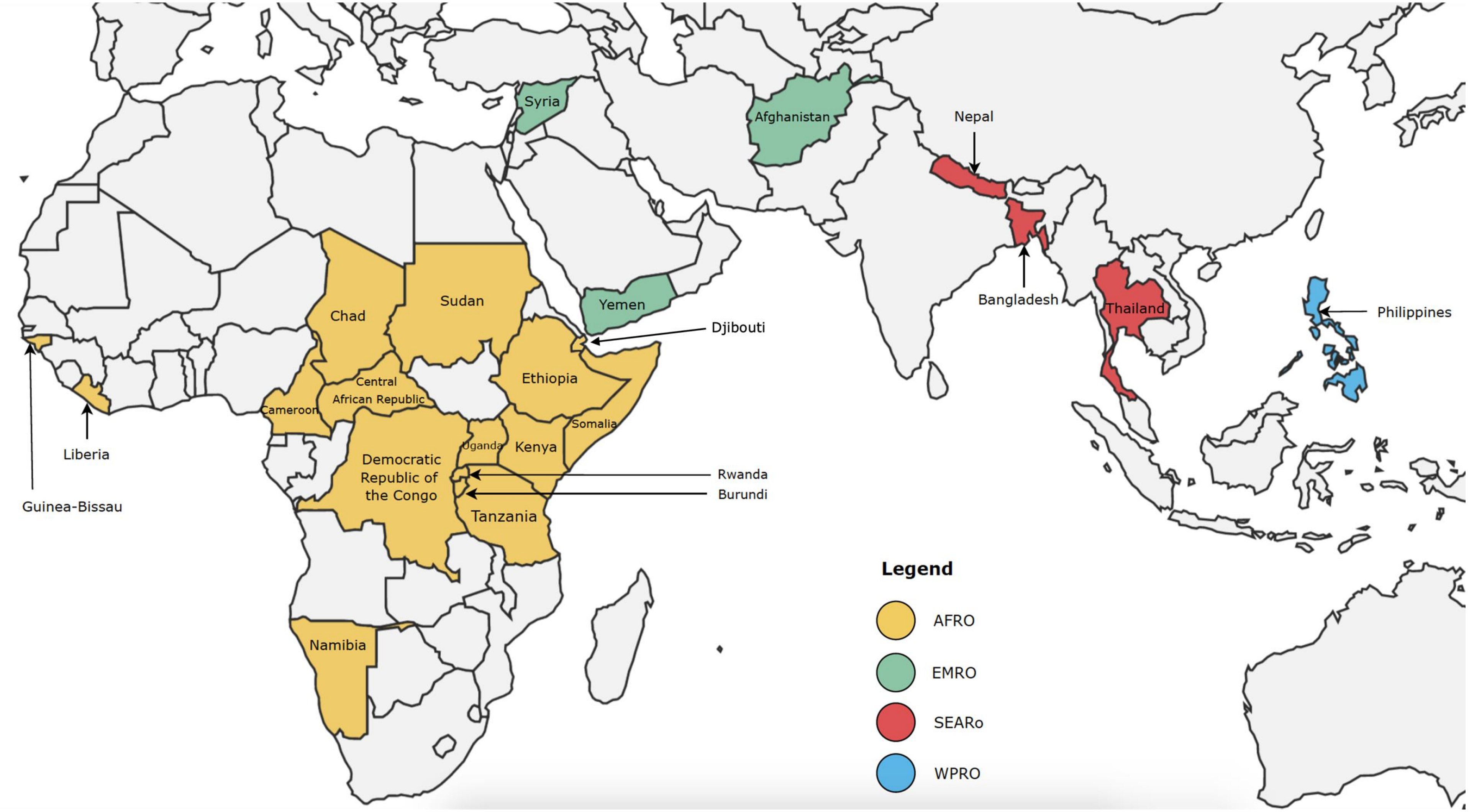

AFRO - African Regional Office (Yellow), EMRO - Eastern Mediterranean Regional Office (Green), SEARO South-East Asian Regional Office (Red), WPRO – Western Pacific Regional Office (Blue). Note: Hershey et al (2011) study includes data on UNHCR refugee camps in the following 16 countries: Banglades h, Burundi, Cameroon, Chad, Democratic Republic of Congo, Djibouti, Ethiopia, Kenya, Namibia, Nepal, Rwanda, Sudan, Tanzania, Thailand, Uganda and Yemen.

Table S1. Pneumonia incidence and proportional morbidity among children in humanitarian emergencies in low- and middle-income countries.

Population	Age groups	Pneumonia incidence rate (episodes per 100 person-years)[*]			Pneumonia proportional morbidity[§] (%)		
		Refugee/IDP camp	Conflict (general)	Natural disaster	Refugee/IDP camp	Conflict (general)	Natural disaster
**Community level**	All children (2m-17y)	-	-	-	-	13% (van Berlaer 2017)<br>9.0% (primary diagnosis)[‖]	-
	Children U5 (2m-4y)	1448 (Summers 2018)<br>73 (Turner C. 2013)[†]<br>50 (non-severe)<br>15 (severe)<br>6 (very severe)<br>22 (radiological end-point)	-	-	-	-	-
	Infants (2-11m)	-	146.0 (Manaseki-Holland 2012)[‡]<br>125.9 (pneumonia)<br>8.0 (severe pneumonia)<br>13.7 (radiology-confirmed)	-	-	-	-
	No disaggregated data for: Young children (1-4y), Older children (5-9y), Adolescents (10-17y).						
**Primary care level**	All children (2m-17y)	-	-	-	-	-	-
	Children U5 (2m-4y)	71.04 (Hershey 2011, Africa)<br>305.4 (Hershey 2011, Asia)	-	-	17% (Hershey 2011)<br>34% (Asia)<br>13% (Africa)	9.7% (Anwar 2016)<br>12.2% (2005-2007)<br>9.9% (2008-2010)<br>8.6% (2011-2013)<br>16.0% (Clarke-Deelder 2019)[¶]<br>21.2% (Bernasconi 2018)	-
	No disaggregated data for: Infants (2-11m), Young children (1-4y), Older children (5-9y), Adolescents (10-17y).						
**Hospital level**	All children (2m-17y)	-	-	-	-	7.2% (Sodemann 2004)<br>10.8% (pre-conflict)	12.45% (van Berlaer 2019)<br>16.4% (Chang 2016, impact)[#]

						22.2% (Meiqari 2018)	22.0% (pre-impact)
						23.4% (Huerga 2009)	19.4% (post-impact)
						48% (Ngoy 2013)	22.3% (Giri 2018)[#]
	Children U5 (2m-4y)	-	-	-	-	9.4% (Birindwa 2020)<br>12.2% pre-PCV13<br>7.1% post-PCV13	18.77% (van Berlaer 2019)
	Infants (2-11m)	-	-	-	-	(Meiqari 2018)<br>34.2% (<6 months)<br>27.7% (6-11 months)	-
	Young children (1-4y)	-	-	-	-	20.6% (Meiqari 2018)	-
	Older children (5-9y)	-	-	-	-	12% (Meiqari 2018)	5.24% (van Berlaer 2019)
	Adolescents (10-17y)	-	-	-	-	6.8% (Meiqari 2018)	

IDP = internally displaced persons; U5 = children under 5 years of age.

[*]Pneumonia incidence rates have been converted to episodes per 1000 person-years to allow comparison between studies.

[†]Pneumonia classified using pre-2014 WHO pneumonia definitions.

[‡]Pneumonia classified using WHO pneumonia and severe pneumonia categories.

[§]Proportional morbidity = pneumonia cases as a proportion of all paediatric cases in community, presenting to outpatient facilities, or admitted to hospital, unless otherwise specified.

[‖]Van Berlaer reported for pneumonia as a primary diagnosis, or as any diagnosis.

[¶]Severe pneumonia, % of all paediatric outpatients.

[#]Pneumonia, % of paediatric medical admissions.

Table S2. Proportional mortality, case fatality rates, and incidence of death from pneumonia among children in humanitarian emergencies in low- and middle-income countries.

Indicator population	Age groups	Pneumonia proportional mortality[*] (%)			Case fatality rate[†] (%)			Incidence of death from pneumonia (deaths per 1000 child-years)[‡]		
		Refugee/IDP camp	Conflict (general)	Natural disaster	Refugee/IDP camp	Conflict (general)	Natural disaster	Refugee/IDP camp	Conflict (general)	Natural disaster
**Community level**	All children (2m-17y)	-	-	-	-	-	-	-	-	-
	Children U5 (2m-4y)	-	8.3% (Robinson 2021)	-	-	-	-	0.5 (Turner C. 2013)	-	-
	Infants (2-11m)	-	-	-	-	-	-	-	4·2 (Manaseki-Holland 2012)	-
	No disaggregated data for: Young children (1-4y), Older children (5-9y), Adolescents (10-17y).									
**Primary care level**	All children (2m-17y)	-	-	-	-	-	-	-	-	-
	Children U5 (2m-4y)	20% (Hershey 2011)<br>20% (Africa)<br>14% (Asia)	-	-	-	-	-	-	-	-
	No disaggregated data for: Infants (2-11m), Young children (1-4y), Older children (5-9y), Adolescents (10-17y).									
**Hospital level**	All children (2m-17y)	-	30.2% (Ngoy 2013)[§]<br>21.8% (Huerga 2009)[§]	-	-	13.1% (Sodemann 2004)<br>17.2% (peace cohort)<br>12% (Huerga 2009)[§]<br>2.1% (Ngoy 2013)[§]<br>0.5% (Meiqari 2018)	-	-	-	-
	Children U5 (2m-4y)	-	-	-	-	12.1% (Zabihullah 2017)<br>4.3% (Rasooly 2020)<br>4.9% (Birindwa 2020)<br>4.8% pre-PCV13<br>4.9% post-PCV13<br>0.7% (Manaseki-Holland 2010)[‖]	-	-	-	-
	Infants (2-11m)	-	-	-	-	Meiqari 2018	-	-	-	-

						0.4% (< 6 months)<br>1.4% (6-11 months)				
	Young children (1-4y)	-	-	-	-	0% (Meiqari 2018)	-	-	-	-
	Older children (5-9y)	-	-	-	-	0% (Meiqari 2018)	-	-	-	-
	Adolescents (10-17y)	-	-	-	-	0% (Meiqari 2018)§	-	-	-	-

Children U5, children under 5 years of age

*Proportional mortality = pneumonia deaths as a proportion of all paediatric deaths in community, or hospital, unless otherwise specified.

†Case fatality rate = pneumonia deaths as a proportion of pneumonia cases.

‡Estimates for pneumonia incidence of death have been converted to deaths per 1000 person-years to allow comparison between studies.

§Under 15 years of age.

‖1-36 months of age.

Table S3. Other key findings from studies included in this review.

Area of interest	Study	Key findings
**Household and other contextual factors associated with pneumonia**	**Hershey et al (2011)**	**Refugee camp size and location**<br>Large refugee camps (≥ 20,000 people) were associated with a significantly increased risk of pneumonia compared to small refugee camps (< 10,000 people) (IRR = 2.07, 95% CI 1.03-4.15).<br>Refugee camps in Asia had a higher risk of pneumonia compared to those in Africa (IRR = 4.52, 95% CI 3.18-6.41) however after adjusting for other camp characteristics, this difference was not significant.<br>**Water and sanitation**<br>Proximity to a water source was associated with an increased risk of pneumonia (IRR = 1.38, 95% CI 1.06-1.81)<br>There was no significant association between the incidence of pneumonia and water quantity, water access, latrine access, latrine coverage, or soap access.<br>**Nutrition standards**<br>There was no significant association between the incidence of pneumonia and global acute malnutrition or ration adequacy<br>**Health service utilisation**<br>A higher number of new patient visits to a health facility was associated with a significantly increased risk of pneumonia (IRR = 1.73, 95% CI 1.26- 2.37) which may reflect increased care-seeking behaviour<br>There was no significant association between the incidence of pneumonia and growth monitoring utilisation
	**Rasooly et al (2020)**	**Factors associated with significantly increased risk of severe pneumonia:**<br>Children aged between 2 and 5 months (OR = 1.44; 95% CI: 1.06–1.95).<br>Residing in rural areas (OR = 2.06, 95% CI: 1.51–1.80)<br>Living more than one hour distance from a health centre (OR = 2.2; 95% CI: 1.2–4)<br>**Factors associated with a significantly decreased risk of severe pneumonia:**<br>Use of kitchens with windows (OR = 0.37; 95% CI: 0.26–0.53).
	**Turner, C. et al (2013)**	**Factors associated with a significantly increased risk of pneumonia:**<br>Neutrophils >7.5 x 10^9/L and CRP ≥ 40mg/L were associated with a significantly increased risk of primary endpoint pneumonia (OR 2.37, 95% CI 1.56–3.59)<br>Maternal age < 18 years was associated with a significantly increased risk of first episode of pneumonia (IRR 1.59, 95% CI 1.12–2.27, p = 0.01)<br>**Factors associated with a significantly decreased risk of pneumonia:**<br>Neutrophils >7.5 x 10^9/L and CRP ≥ 40mg/L were associated with a significantly decreased risk of other infiltrate pneumonia (OR 0.54, 95% CI 0.36–0.81)<br>Distance from stove to bed was associated with a significantly decreased risk of primary endpoint pneumonia (IRR 0.89, 95% CI 0.80–0.99, p = 0.03)<br>Number of rooms was associated with a significantly decreased risk of first episode of pneumonia (IRR 0.85, 95% CI 0.77–0.95, p = 0.004)<br>There was no significant association between the incidence of pneumonia and season of birth, the distance from the infant's sleeping area and the household stove, birth weight, or the number of people sleeping in the same bed as the infant
**Risk factors for death from pneumonia**	**Birindwa et al (2020)**	**Risk of death among hospitalised children aged <5 years with ALRI was significantly increased in those with:**<br>• Malnutrition (adjusted OR 8.03, 95% CI 3.18–20.28)<br>• Congenital diseases (adjusted OR 9.85, 95% CI 1.76–55.28)
	**Sodemann et al (2004)**	**Factors associated with a lower case fatality risk for war versus peace:**<br>Mother did not have schooling (age-group adjusted OR 0.27, 95% CI 0.10–0.73)<br>House roof was made of straw (rather than zinc tiles) (OR 0.20, 95% CI 0.06–0.61)<br>House did not have electricity (OR 0.55, 95% CI 0.33–0.91)<br>Family did not have a TV set (OR 0.56, 95% CI 0.35–0.89)

	**Zabihullah et al (2017)**	**The risk of death from pneumonia among children aged <5 years was significantly increased in:**<br>• Age <1 month compared to age ≥12 months (adjusted OR 13.1, 95% CI 3.71–46.5, p <0.01)<br>• Malnutrition (adjusted OR 2.06, 95% CI 1.22–3.49, p <0.01)<br><br>The risk of death from pneumonia among children aged <5 years was significantly decreased in those who had received the BCG vaccine compared to those who had not received the BCG vaccine (adjusted OR 0.47, 95% CI 0.25–0.88, p = 0.02)<br>Compared to children aged ≥12 months, children aged 1-11 months old were not at a significantly increased risk of death from pneumonia (adjusted OR 1.59, 95% CI 0.72–3.49, p = 0.24).<br>Compared to children whose mothers were literate, children whose mothers were illiterate were not at a significantly increased risk of death from pneumonia (adjusted OR 0.62, 95% CI 0.26–1.45, p = 0.27).
**Aetiology**	**Turner, C. et al (2012) and Turner, C. et al (2013)**	**Viral detection via PCR on nasopharyngeal (NPA) samples:**<br>Viruses were detected in 61.3% of pneumonia episodes in a birth cohort of child refugees who were followed for 2 years; 16.3% were positive for two viruses and 1.3% were positive for three viruses.<br>RSV was the most commonly detected virus and was detected in 33.9% of pneumonia cases (and 54.2% of pneumonia in children aged < 2 months of age), with marked seasonality - 80% of pneumonia episodes that occurred every October and November during the study period were associated with RSV.<br>Other common viruses detected were adenovirus, influenza A and B, and human metapneumovirus (hMPV).
	**Turner, P. et al (2013)**	**Viral detection via PCR on nasopharyngeal (NPA) samples:**<br>Viruses were detected in 53.7% of NPA samples; 4.0% of specimens were positive for multiple viruses.<br>The most commonly detected virus was RSV (24.9% of NPA samples), followed by adenovirus (18.8%), influenza A (8.2%), hMPV (4.7%), and influenza B (1.4%).<br>RSV, influenza A and B, and hMPV were detected in the wet and cool seasons (June – October and November–February respectively). In contrast, adenovirus was detected all year and peaked in the late cold and hot seasons (March–May).
	**Zabihullah et al (2017)**	**Detection of *Streptococcus pneumoniae* in nasopharyngeal samples:**<br>*S. pneumoniae* was detected in 38.0% of nasopharyngeal samples from children aged < 5 years; however 49.9% of children in the study were not swabbed due to severe disease. severity.<br>*S. pneumoniae* was detected in 35.9% of children who received antibiotics prior to admission and 48.8% who did not.<br>Pneumococcal serotypes targeted by the PCV-7, PCV-10, and PCV-13 vaccines were found in 39.5%, 39.5% and 46.8% of samples respectively. 16.9% of positive samples contained multiple serotypes.
**Prevention**	**Bernasconi et al (2018)**	Baseline immunisation rates were 39.7% among children aged 2-59 months old (immunization details not described)
	**Meiqari et al (2017)**	MSF-OCA administered a total of 23,925 vaccine doses in January-May 2014, 6,591 doses in October-December 2015, and 41,111 doses in January-September 2016.<br>Reported vaccines included BCG for all ages, hepatitis B at birth, oral or intramuscular polio, measles or measles/mumps/rubella, and diphtheria, pertussis, and tetanus plus hepatitis B plus haemophilus influenza type B.
**Diagnosis and treatment**	**Bernasconi et al (2018)**	Baseline adherence to IMCI guidelines was poor. 88/599 (14.7%) children had respiratory rate documented despite over half presenting with cough or respiratory symptoms. Antibiotic usage decreased following introduction of an electronic decision-making tool, suggesting more rational use of antibiotics, however specific data on pneumonia treatment was not reported.

	**Clarke-Deelder et al (2019)**	Of the IMCI-determined severe pneumonia (or other severe respiratory infection), 52% were diagnosed with pneumonia or other respiratory infection and 19% were diagnosed with severe pneumonia or other severe respiratory infection by their healthcare provider. 69% of severe pneumonia cases were prescribed antibiotics<br>29.6% of severe pneumonia patients were treated in hospital, 7.4% were treated in a referral health centre, and 63% were treated in other health centres.<br>Among the severe pneumonia patients, 34.4% of their healthcare providers had training on IMCI protocols while 65.6% of providers had not received such training. Patients were most commonly cared for by a nurse (72.5%), followed by a doctor (22.8%) and other healthcare provider (4.8%)
	**Summers et al (2018)**	Of 1,017 children aged 6-59 months with acute respiratory infection, 64.9% were treated through a formal health system, 18.0% received other treatment (treatments outside of the formal health system including local pharmacies, community or traditional healers, and other unspecified treatments), and 17.1% received no treatment
	**Turner, C. et al (2012)**	12 clinical signs were assessed for any significant **association with RSV detection**<br>In univariate analysis, 5 of these clinical signs were significantly associated with RSV-positive pneumonia: fever on admission, tachycardia, hypoxia, chest in-drawing and bilateral crepitations or wheeze. On multivariate analysis only fever on admission, tachycardia, and bilateral crepitations or wheeze were significant<br>Clinical sign with the highest sensitivity was bilateral chest signs (crepitations or wheeze, 75.0%), followed by fever on admission (33.1%) & tachycardia (18.1%)<br>The clinical sign with the highest specificity was tachycardia (88.4%), followed by fever on admission (74.4%), and bilateral chest signs (36.8%)
**Comparisons with non-crisis-affected populations**	**Chang et al (2016)**	Proportional morbidity of pneumonia (out of all paediatric infectious disease admissions) rose from 40.0% during the pre-impact phase to 57.3% during the impact phase of typhoon Haiyan. During the post-impact phase, proportional morbidity of pneumonia decreased to 34.1% however this was due to an overall decrease in paediatric infectious disease admissions; the number of paediatric pneumonia cases remained the same during the impact and post-impact phases
	**Giri et al (2018)**	During a fifteen-week period following the 2015 Nepal earthquake, rates of pneumonia were significantly higher in admitted children from affected districts compared to those from non-affected districts (26.6% vs 17%, p <0.001). However, when comparing children whose family and/or house were substantially affected by the earthquake and those whose family and/or house were not, there was no significant difference in pneumonia rates
	**Sodemann et al (2004)**	Sodemann et al compared paediatric patients hospitalised during a period of war in Guinea-Bissau ('war cohort') with paediatric patients hospitalised in the 12-month period preceding the conflict ('peace cohort'), and found no significant difference in case-fatality rates for pneumonia; the case-fatality risk for war versus peace odds ratio was 0.72 (95% CI 0.46–1.13)
	**van Berlaer et al (2017)**	van Berlaer et al compared Syrian children living in town versus IDP camps and identified a lower rate of infectious diseases in children from the camps (65.1% vs 57.1%, p = 0.038). However, on multiple logistic regression analysis this difference was not significant (OR 1.01, 95% CI 0.709-1.438)

IRR, incidence rate ratio; OR, odds ratio; 95% CI, 95% confidence interval; CRP, C-reactive protein; BCG vaccine, Bacillus Calmette–Guérin vaccine; NPA, nasopharyngeal aspirate; PCV, pneumococcal conjugate vaccine; MSF-OCA, Médecins Sans Frontières Operational Centre Amsterdam; IMCI, Integrated Management of Childhood Illness.
